# EGF-mediated reduced miR-92a-1-5p controls HTR-8/SVneo cell invasion through activation of MAPK8 and FAS which in turn increase MMP-2/-9 expression

**DOI:** 10.1038/s41598-020-68966-4

**Published:** 2020-07-23

**Authors:** Ankita Malik, Rahul Pal, Satish Kumar Gupta

**Affiliations:** 1 Reproductive Cell Biology Lab, National Institute of Immunology, Aruna Asaf Ali Marg, New Delhi, 110 067 India; 2 Immunoendocrinology Lab, National Institute of Immunology, Aruna Asaf Ali Marg, New Delhi, 110 067 India

**Keywords:** Cell invasion, miRNAs

## Abstract

The members of human miR-17-92 cluster are implicated in several cancers and are known to increase cancer cells invasiveness. The present study reports reduced expression of miR-92a-1-5p in EGF treated HTR-8/SVneo trophoblastic cells by NGS and qRT-PCR. Overexpression of miR-92a-1-5p led to significantly reduced EGF-mediated HTR-8/SVneo cells invasion. MAPK8 and FAS were predicted to be miR-92a-1-5p targets, and confirmed to be reduced by qRT-PCR and Western blotting in trophoblast cells overexpressing miR-92a-1-5p. The binding of miR-92a-1-5p to MAPK8 and FAS 3′-UTR was confirmed by Luciferase reporter assay and Rescue assay. EGF increases MMP-2 & MMP-9 expression and reduces TIMP1 expression in HTR-8/SVneo cells. Inhibition of MAPK8 (by SP600125) reduced EGF-mediated MMP-9/TIMP1 ratio and invasion. Similarly, silencing of FAS by siRNA reduced EGF-mediated MMP-2/TIMP1 ratio and invasion. Treatment of HTR-8/SVneo cells with STAT1/3 inhibitors or siRNAs led to loss of EGF-mediated reduction in miR-92a-1-5p levels. Inserting the predicted binding sites of STAT3 present in promoter region of miR-92a-1-5p upstream of Luciferase promoter reduced its expression in presence of STAT3 expression vector. Thus, EGF leads to reduced miR-92a-1-5p expression which may be regulated by STAT1/STAT3 and controls HTR-8/SVneo cells invasion by targeting MAPK8 and FAS, which in turn increases MMP-2/MMP-9 expression.

## Introduction

During pregnancy, the fine balance of pro- and anti- invasion growth factors and cytokines at feto-maternal interface is crucial for proper implantation and placentation. These growth factors and cytokines activate various signaling pathways and regulate the expression of effector proteins such as matrix metaloproteinases (MMPs), tissue inhibitors of MMPs (TIMPS), integrins, cadherins etc. The effector proteins are responsible for degradation of extracellular matrix, establishment of contact between extravillous trophoblast cells (EVTs) and maternal decidual cells, spiral artery remodeling and migration of EVTs resulting in placentation. Poor EVT invasion is associated with preeclampsia, fetal growth restriction, and early and late recurrent miscarriages^1^. Recently, microRNAs (miRNAs) have acquired importance amongst the regulatory factors influencing the trophoblast proliferation, syncytialization and invasion^2^. These miRNAs (19–22 nucleotides long) bind to complementary regions in the 3′-UTR of target mRNAs through seed sequences and modulate gene expression by either mRNA degradation or its translational inhibition. The human placenta expresses a number of ubiquitous miRNAs, as well as some which are only specific to the trophoblasts^3^. They are involved in key placental functions, and differences in miRNA expression profiles have been reported in placentas obtained from healthy and compromised pregnancies^4,5^.

The circulating levels of three out of six members of miR-17-92 cluster (miR-18a, miR-19b1, and miR-92a) are reported to be significantly lower in serum from preeclampsia patients as compared to normal controls during early gestation (15–18 weeks) and at term^6^. The human miR-17-92 cluster is oncogenic (identified as Oncomir -1)^7,8^ and contrasting roles for the miRNAs of this cluster are reported. The miRNA miR-17-92 cluster encoding gene (*C13orf25*) is located at the open reading frame of human chromosome 13, and generates a polycistronic primary transcript with six tandem stem-loop hairpin structures. Cleavage of the polycistronic primary transcript yields the six mature miRNAs (miR-17, miR-18, miR-19a, miR-20, miR-19b, and miR-92a-1)^9,10^. This cluster plays crucial role during tumor progression in various human cancers, and its expression is reported to be controlled by STAT3^10,11^. The miR-92a-1 stem loop sequence (Fig. S1) from the miR-17-92 cluster encoding gene, yields two mature miRNAs—miR-92a-1-5p and miR-92a. The miR-92a-1-5p (previously known as miR-92a-1*) is the least described among the two. Whereas, miR-92a (now known as miR-92a-3p) is reported to be overexpressed in various types of tumors, and increases cell proliferation and invasion^12–14^.

This study attempted to identify differentially expressed miRNAs and their target genes during epidermal growth factor (EGF)-mediated HTR-8/SVneo trophoblastic cells invasion through next generation sequencing (NGS). Differentially expressed miRNAs and mRNAs identified following NGS of EGF treated HTR-8/SVneo cells as compared to untreated control cells were confirmed by quantitative real-time PCR (qRT-PCR). This study reports that the expression of miR-92a-1-5p is reduced significantly during EGF-mediated HTR-8/SVneo cell invasion and its overexpression leads to reduced invasion in EGF treated HTR-8/SVneo cells, which may be regulated by STAT1 and STAT3. Overexpressed miR-92a-1-5p in these cells down regulated FAS (CD95) and MAPK8 (JNK1) expression. The cell invasion is reduced through FAS-mediated decrease in MMP-2/TIMP1 and MAPK8-mediated decrease in MMP-9/TIMP1 ratios.

## Results

### microRNA expression profile in EGF treated HTR-8/SVneo cells

**Figure 1:**
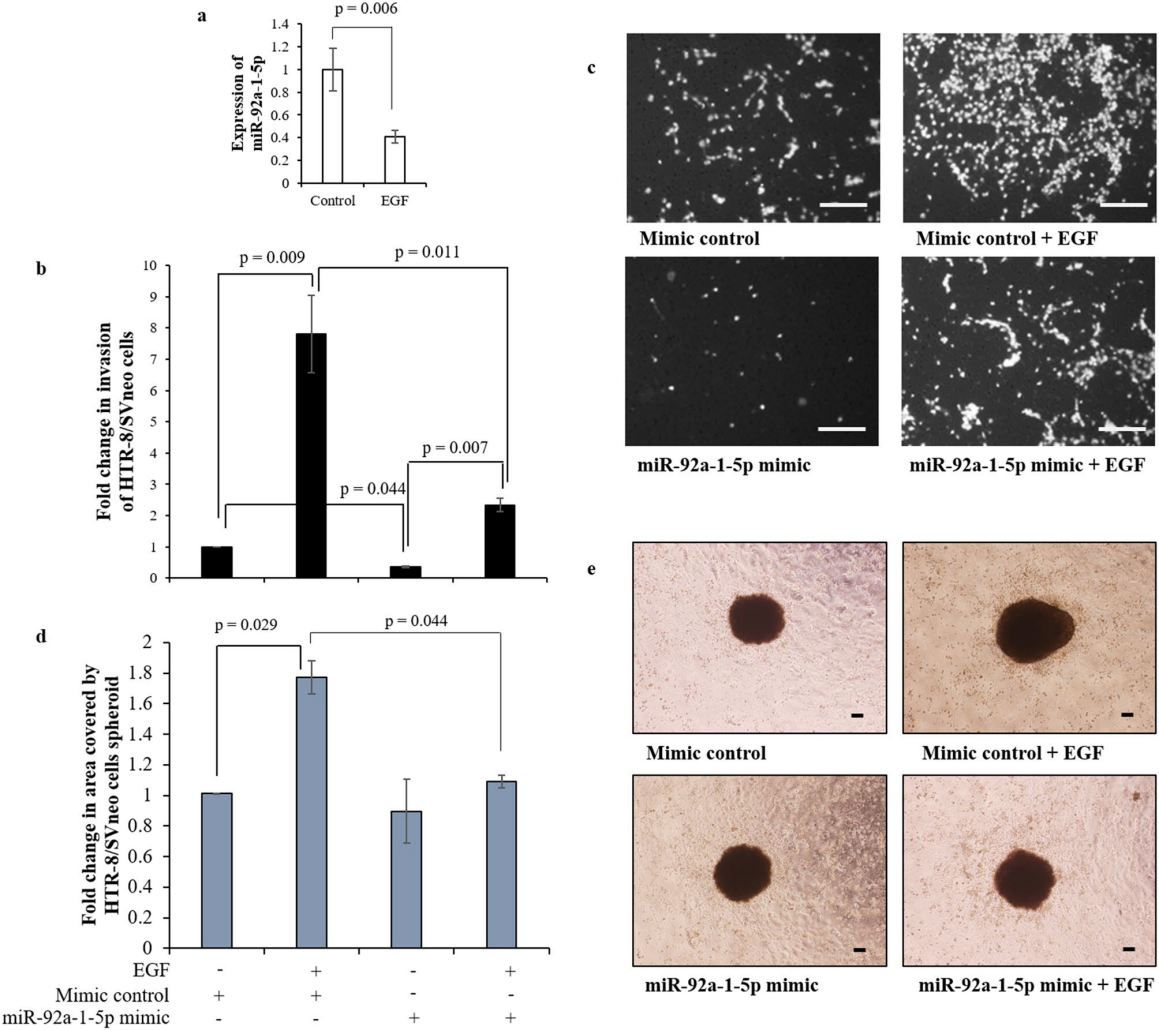
miR-92a-1-5p expression and effect of its mimic during EGF-mediated HTR-8/SVneo cell invasion. (**a**) qRT-PCR for the expression of miR-92a-1-5p relative to miR-191 in HTR-8/SVneo cells with or without EGF treatment. Data in (a) is mean ± SEM of three independent experiments performed in triplicates. (**b**) Fold change in invasion of EGF treated and untreated (24 h) miR-92a-1-5p mimic transfected HTR-8/SVneo cells as compared to EGF untreated control mimic transfected HTR-8/SVneo cells in transwell invasion assay. Data in (**b**) is mean ± SEM of three independent experiments performed in duplicates. (**c**) Representative photographs of invading cells in various treatment groups on 0.8 µm pore size transwell membranes as observed under microscope after processing for invasion assay. Scale bars represent 5 µm. (**e**) Fold change in the area covered by individual scrambled siRNA-transfected HTR-8/SVneo spheroids and miR-92a-1-5p mimic transfected HTR-8/SVneo spheroids, respectively, in the presence/absence of EGF after 24 h. Each bar represents a relative area of spreading after normalization with untreated scrambled siRNA-transfected HTR-8/SVneo spheroids. (**c**) Representative photographs of the spheroids are appended. Scale bar represents 20 μm. Values are expressed as mean ± SEM of three independent experiments.

RNA isolated from HTR-8/SVneo cells treated with or without 10 ng/ml EGF for 24 h was used to perform NGS. Following deep sequencing, array platform used was in accordance with miRBase Release 21. Out of a total number of 2,588 mature *Homo sapiens* miRNAs; 1,609 mature miRNA’s were detected in both EGF treated and untreated samples. Differential miRNA expression was analyzed by comparing two groups: EGF treated (24 h) and the untreated control. The statistically significant differentially expressed miRNAs between the treated and untreated groups were determined by one way ANOVA test. A *p* value < 0.05 was considered statistically significant. One hundred and seventy-eight miRNAs were found to be significantly differentially expressed in EGF treated sample as compared with the untreated control (Accession Number—GSE124585). Further, 62 out of the 178 differentially up-/down-regulated miRNAs showed ≥ 1.5-fold change (Fig. S2). Out of the differentially expressed miRNA, miR-92a-1-5p and miR-19a-5p belong to miR-17-92 cluster (C13orf25) and both are reduced after EGF treatment in HTR-8/SVneo cells. Reduced expression of miR-92a-1-5p and miR-19a-5p in HTR-8/SVneo cells treated with EGF was further confirmed by qRT-PCR (Fig. 1a, Fig. S3).

### miR-92a-1-5p mimic transfected HTR-8/SVneo cells show reduced EGF-mediated invasion

The relevance of miR-92a-1-5p and miR-19a-5p was investigated by performing invasion assay with cells transfected with their individual mimics and control mimic with or without EGF (10 ng/mL) treatment. Significantly reduced basal invasiveness as compared to control mimic transfected cells was observed in HTR-8/SVneo cells transfected with miR-92a-1-5p mimic (Fig. 1b,c). Further, significant reduction in EGF-mediated increased HTR-8/SVneo cell invasiveness was observed in miR-92a-1-5p mimic transfected HTR-8/SVneo cells as compared to EGF treated control mimic transfected cells (Fig. 1b,c). In an additional experiment, significant reduction in area covered (spreading) was observed by spheroids formed from miR 92a-1-5p mimic transfected HTR-8/SVneo cells treated with EGF in co-culture experiment with Ishikawa cells monolayer, as compared to area covered by spheroids formed from control mimic transfected HTR-8/SVneo cells treated with EGF (Fig. 1d,e). Inhibition of miR-92a-1-5p expression in HTR-8/SVneo cells by using specific inhibitor led to an increase in basal invasiveness of HTR-8/SVneo cells (~ 2.2 fold, Fig. S4). When miR-92a-1-5p inhibitor transfected cells were subsequently treated with EGF, a significant (*p* = 0.022) increase in the HTR-8/SVneo cells invasion was observed as compared to miR-92a-1-5p inhibitor transfected cells without EGF treatment (Fig. S4). Further, no significant decrease in the invasion of the cells transfected with miR-92a-1-5p inhibitor and treated with EGF was observed as compared to inhibitor control treated with EGF (Fig. S4).

However, cells transfected with miR-19a-5p mimic still showed significantly increased invasion subsequent to EGF treatment as compared to cells without EGF treatment. Increased expression of miR-19a-5p failed to inhibit/reduce EGF-mediated increased HTR-8/SVneo cells invasiveness (Fig. S3).

### MAPK8, TCF7, CD151 and FAS are predicted targets of miR-92a-1-5p

RNA isolated from HTR-8/SVneo cells treated with or without 10 ng/ml EGF for 24 h was outsourced to Bionivid Pvt. Ltd. for RNA quality check followed by mRNA NGS. Differential mRNA expression analysis was performed by comparing the EGF treated and EGF untreated groups and the statistical significance of differentially expressed mRNAs between the two groups was determined by one way ANOVA. A *p* value ≤ 0.05 was considered as statistically significant. A total of 9,568 genes were detected in NGS out of which 1,051 were found to be significantly differentially expressed in 24 h EGF treated sample as compared with the 24 h untreated control (Accesion Number—GSE124586). Out of the 1,051 differentially expressed genes (DEGs), 209 genes had more than two fold difference in expression as compared to the untreated control; 96 genes of these 209 genes were increased in abundance and 113 showed reduced expression (Fig. S5).

Using miRNA target prediction tools like TargetScan, microRNA.org, miRDB, miRNet, miRanda and miRTarBase, *in-silico* analysis of various EGF-induced differentially expressed genes was performed to identify the targets of miR-92a-1-5p. Out of the differentially expressed genes, *MAPK8*, *TCF7*, *CD151* and *FAS* were found to be predicted targets of miR-92a-1-5p (Fig. S6). Predicted consequential pairing (from TargetScan, miRanda and miRTarBase) showed interaction of miR-92a-1-5p with 3′-UTR of the target mRNA of *MAPK8*, *TCF7*, *CD151* and *FAS* (Fig. S6). The complementary sequence site of *MAPK* and *FAS* is 7mer-A1 type and in case of *TCF7* and *CD151* it is 7mer-m8 type (Fig. S6).

### Transfection of HTR-8/SVneo cells with miR-92a-1-5p mimic led to reduced expression of MAPK8 and FAS

**Figure 2:**
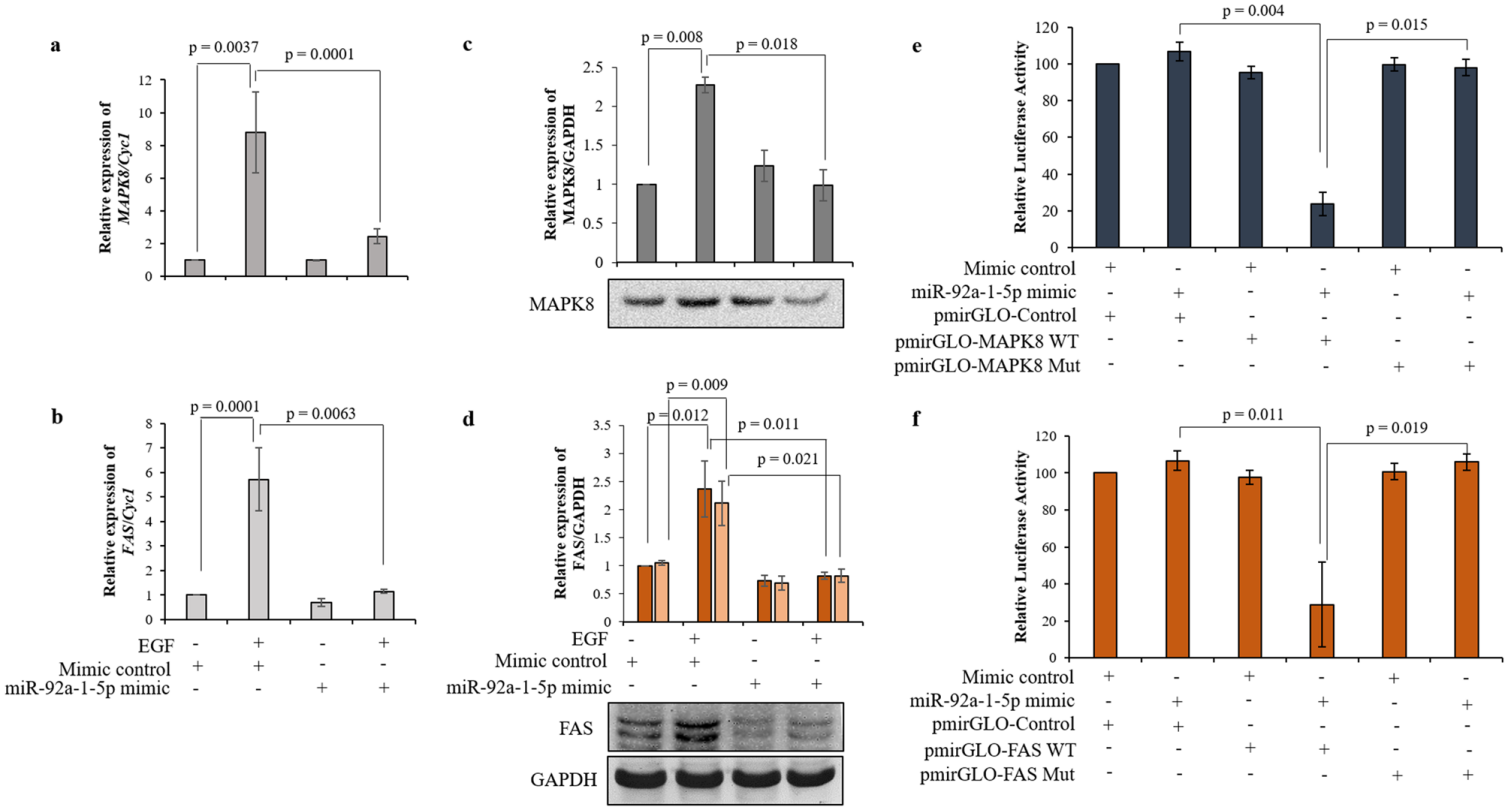
Effect of EGF treatment of miR-92a-1-5p mimic transfected HTR-8/SVneo cells on MAPK8, and FAS expression. Relative expression of *MAPK8* (**a**) and *FAS* (**b**) taking *Cyc1* as internal control in miR-92a-1-5p mimic transfected HTR-8/SVneo cells with or without treatment with EGF as compared to control mimic transfected cells without EGF treatment. Data is represented as mean ± SEM of three independent experiments performed in triplicates. Densitometric profiles of MAPK8 (**c**) and FAS (**d**) with respect to GAPDH as loading control from cell lysates of miR-92a-1-5p mimic transfected HTR-8/SVneo cells treated with or without EGF for 24 h as compared to control mimic transfected cells without EGF treatment. Representative cropped blots from one of the three independent experiments are appended below. The values are expressed as mean ± SEM of three independent experiments. Uncropped blots from three independent experiments are shown in Supplementary Fig. S8 Wild type (WT) as well as mutated *MAPK8* and *FAS* complementary binding sites corresponding to miR-92a-1-5p seed sequence were synthesized and cloned downstream of the firefly luciferase gene under the control of the PGK promoter in pmirGLO Dual-Luciferase vector. (**e**) and (**f**) show the relative luciferase activity determined from HEK-293T cells co-transfected with clones possessing the WT or mutant binding site for miR-92a-1-5p corresponding to *MAPK8* and *FAS* respectively in pmirGLO Dual-Luciferase vector with control or miR-92a-1-5p mimic, as indicated. The data are represented as the mean of four experiments ± S.E.M. performed in duplicates.

To confirm that expression of MAPK8, TCF7, CD151 and FAS is controlled by miR-92a-1-5p during EGF-mediated increased invasiveness of HTR-8/SVneo cells, cells were transfected with control or miR-92a-1-5p mimic and were treated with or without EGF (10 ng/mL) for 24 h. After 24 h EGF treatment, RNA was isolated and processed for qRT-PCR or cell lysates were processed to study the expression of MAPK8, TCF7, CD151 and FAS at protein levels by Western blot. Treatment of HTR-8/SVneo cells with EGF significantly increased transcript levels of *MAPK8* (Fig. 2a), *FAS* (Fig. 2b), *CD151* (Fig. S7) and *TCF7* (Fig. S7). A significant reduction in expression of *MAPK8*, *FAS* and *CD151* was observed in EGF treated miR-92a-1-5p mimic transfected cells as compared to EGF treated control mimic transfected cells (Fig. 2a,b, Fig S7). However, no significant changes in the expression levels of *TCF7* was observed in EGF treated miR-92a-1-5p mimic transfected cells as compared to EGF treated control mimic transfected cells in qRT-PCR (Fig. S7). Expression of MAPK8 and FAS at protein level corroborated with transcript results and were significantly reduced in EGF treated miR-92a-1-5p mimic transfected cells as compared to EGF treated control mimic transfected cells (Fig. 2c,d, Fig. S8). FAS appeared as two bands in Western blots, which may be due to splicing variants being generated in different cell types or due to proteolytic cleaving/deletion of death and transmembrane domains encoding *FAS* sequences^15^ in trophoblast cells. Further, significant change in the expression of CD151 was not observed in miR-92a-1-5p mimic transfected HTR-8/SVneo cells treated with EGF as compared to EGF treated control mimic transfected cells (Fig. S7).

To evaluate whether MAPK8 and FAS are direct targets of miR-92a-1-5p, short nucleotides with the predicted wild type (WT) and mutated (Mut) complementary sequence for both *MAPK8* and *FAS* (table S1) were synthesized and cloned downstream of firefly Luciferase in pmirGLO Dual-Luciferase reporter vector and the effect of miR-92a-1-5p overexpression on luciferase activity evaluated. When the WT *MAPK8* and *FAS* were introduced into HEK-293T cells along with miR-92a-1-5p mimic, it exhibited a significant reduction in the luciferase activity, but the mutation in the binding site in the 3′-UTR of both *MAPK8* and *FAS* suppressed the inhibition of luciferase activity caused by miR-92a-1-5p overexpression (Fig. 2e,f). When WT *MAPK8* and Mut *MAPK8* were co-transfected along with miR-92a-1-5p mimic, luciferase expression was rescued by Mut *MAPK8* (with four mutated nucleotides in miRNA binding complementary sequence to seed sequence, table S1) by ~ 38% (Fig. S9. Similarly, WT *FAS* and Mut *FAS* co-transfection along with miR-92a-1-5p mimic also led to the rescue of luciferase expression by Mut *FAS* (with three mutated nucleotides in miRNA binding complementary sequence to seed sequence, table S1) by ~ 19% (Fig. S9. Thus, miR-92a-1-5p inhibits MAPK8 and FAS expression through direct interaction with the predicted complementary sequence present in their 3′-UTR.

### SP600125 (MAPK8/JNK1 inhibitor) inhibits EGF-mediated HTR-8/SVneo cell invasion and expression of MMP-2 and -9

**Figure 3:**
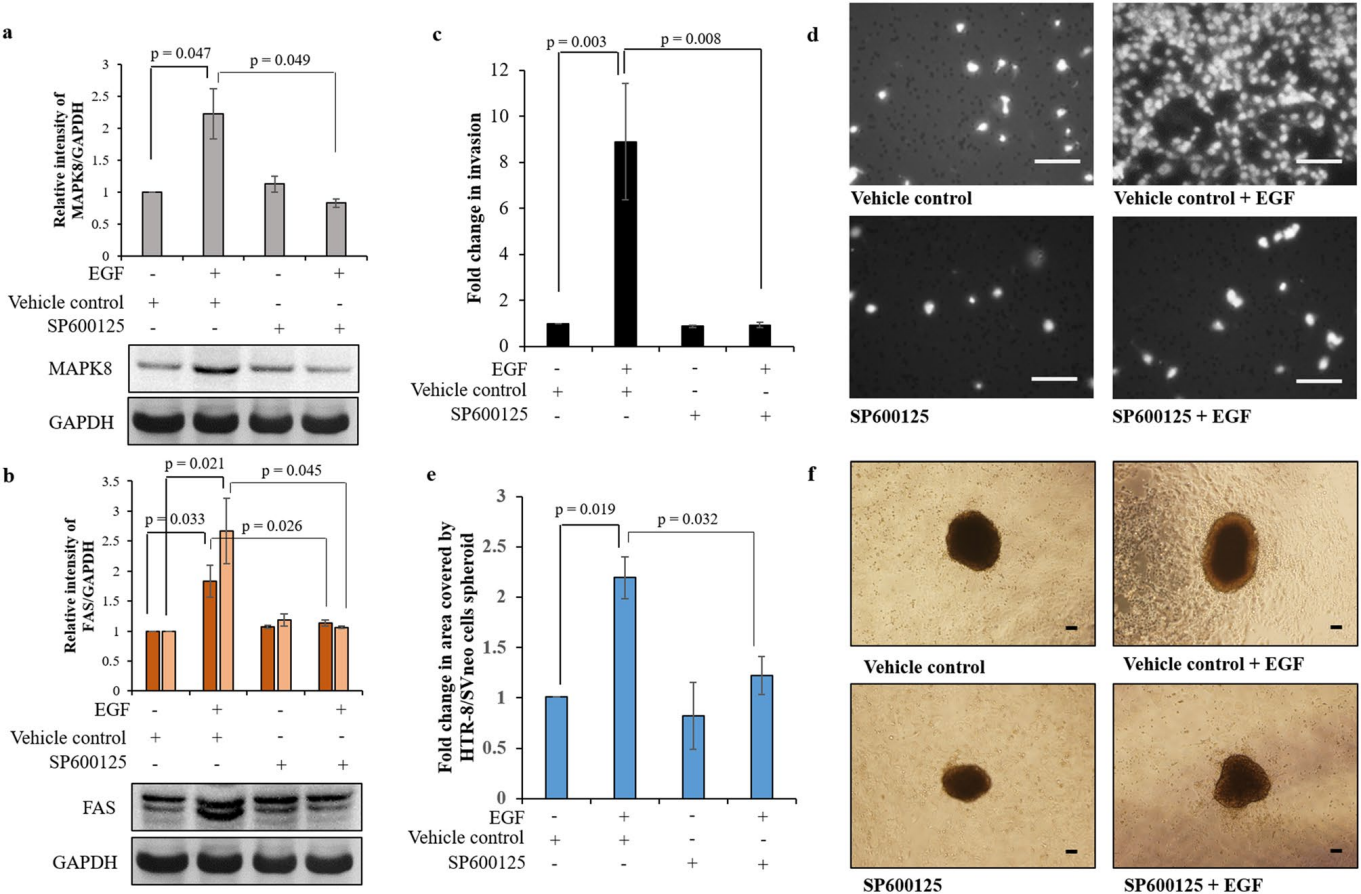
MAPK8 inhibitor reduced EGF-mediated increased FAS expression and HTR-8/SVneo cell invasion. Densitometric profile of MAPK8 (**a**) and FAS (**b**) with respect to GAPDH as loading control in SP600125 pre-treated HTR-8/SVneo cells with or without EGF treatment as compared to control cells without EGF treatment. Representative cropped blots for MAPK8 (**a**), FAS (**b**) and GAPDH from one of the three independent experiments are appended below respective panels. Data in (**a**) and (**b**) is expressed as mean ± SEM of three independent experiments. Uncropped blots from three independent experiments are shown in Supplementary Fig. S10. (**c**) Fold change in invasion of SP600125 pre-treated HTR-8/SVneo cells which were subsequently treated with and without EGF for 24 h as compared to control cells without EGF treatment. Data in (**c**) are mean ± SEM of three independent experiments performed in duplicates. (**d**) Representative photographs of invading cells in various treatment groups on 0.8 µm pore size transwell membranes as observed under microscope after processing for invasion assay. Scale bars represent 5 µm. (**e**) Fold change in the area covered by individual SP600125 pre-treated HTR-8/SVneo spheroids and vehicle control pre-treated HTR-8/SVneo spheroids, respectively, in the presence/absence of EGF after 24 h. Each bar represents the relative area of spreading after normalization with untreated vehicle control HTR-8/SVneo spheroids. **(f**) Representative photographs of the spheroids are appended. Scale bar represents 20 μm. Values are expressed as mean ± SEM of three independent experiments.

To establish the role MAPK8/JNK1 during trophoblast invasion, MAPK8 activation was inhibited by pre-treatment of HTR-8/SVneo cells with SP600125 (25 µM) and cells were used to study their invasion in presence or absence of EGF. HTR-8/SVneo cells pre-treatment with SP600125 led to significant decrease in the EGF-mediated increased expression of MAPK8 as compared to its expression in EGF treated cells without SP600125 pre-treatment (Fig. 3a, Fig. S10). Concomitant significant reduction in FAS activation was also observed in SP600125 pre-treated HTR-8/SVneo cells which were subsequently treated with EGF as compared to EGF treated vehicle control cells (Fig. 3b, Fig. S10). Inhibition of MAPK8 activation led to significantly reduced EGF-mediated increased HTR-8/SVneo cell invasion (Fig. 3c,d). However, no significant effect on HTR-8/SVneo cells basal invasiveness was observed after inhibition of MAPK8 (Fig. 3c,d). Additionally, SP600125 pre-treatment also significantly reduced EGF-mediated HTR-8/SVneo spheroid cells spreading in a co-culture experiment as compared to EGF treated vehicle control cells (Fig. 3e,f).

**Figure 4:**
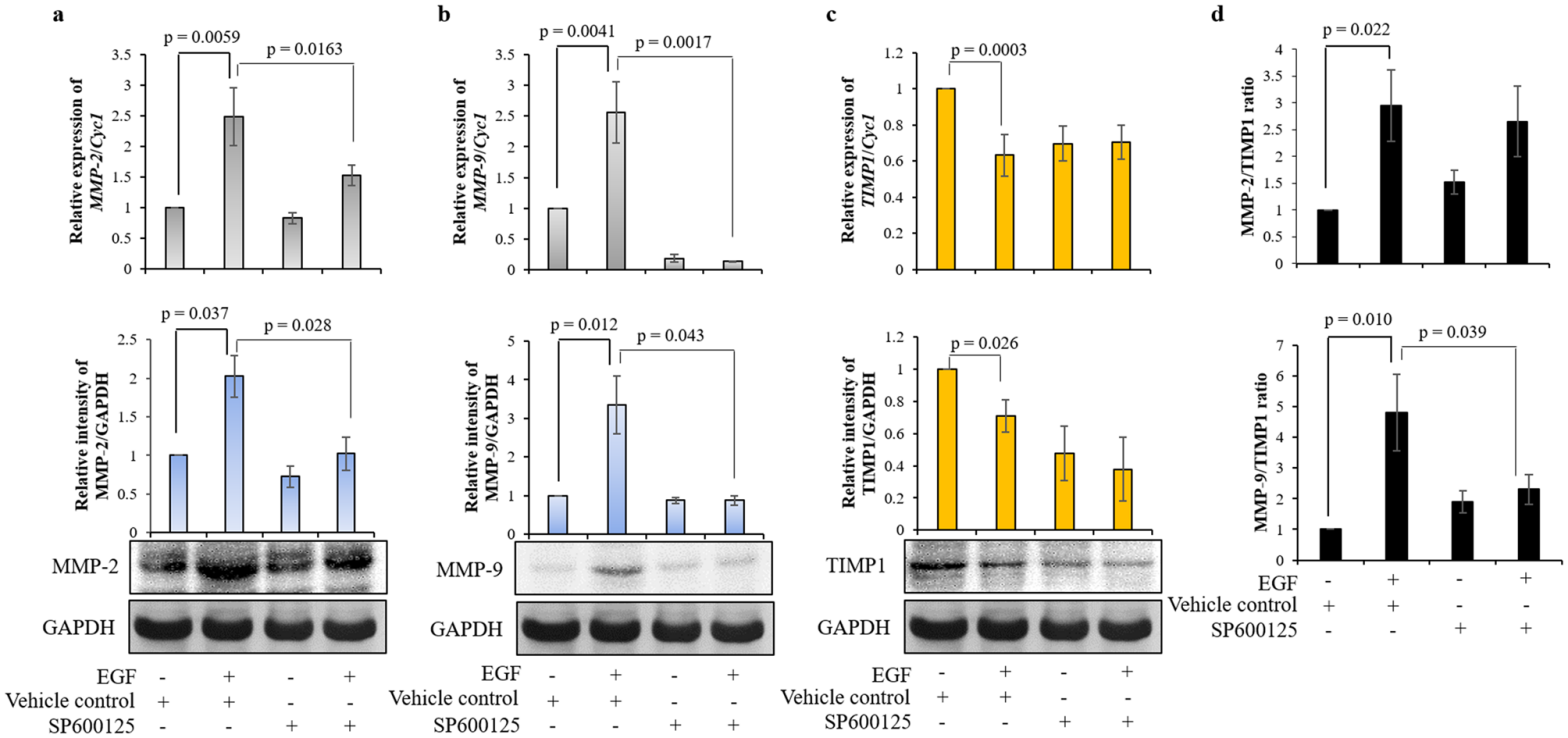
MAPK8 inhibitor reduces EGF-mediated HTR-8/SVneo cell invasion by inhibiting activation of MMP-2 and MMP-9. Relative expression profiles at transcript and protein levels of MMP-2 (**a**), MMP-9 (**b**) and TIMP1 (**c**) with respect to *Cyc1* and GAPDH respectively as loading controls in SP600125 pre-treated HTR-8/SVneo cells which were subsequently treated with and without EGF for 24 h as compared to control cells without pre-treatment with SP600125 and without EGF treatment. Representative cropped blots from one of the three independent experiments are appended below. The transcript data is shown as mean ± SEM of three independent experiments performed in triplicates and protein expression data is shown as mean ± SEM of three independent experiments. (**d**) Ratio of protein levels of MMP-2/TIMP1 and MMP-9/TIMP1. Uncropped blots from three independent experiments are shown in Supplementary Fig. S10.

To delineate the possible effector molecules regulated by MAPK8, which may be responsible in modulating invasion of EGF-treated HTR-8/SVneo cells, the status of various MMPs and TIMPs was studied in the cells subsequent to treatment with or without EGF (24 h). Out of 10 MMPs that were evaluated, treatment with EGF significantly increased the expression of MMP-2 and MMP-9 in HTR-8/SVneo cells (table S2). Whereas, expression of only TIMP1 was significantly reduced out of the 4 TIMPS studied (table S2). Transcript and protein levels of MMP-2 and MMP-9 in SP600125 pre-treated cells with subsequent EGF treatment were significantly reduced as compared to vehicle control cells which were treated with EGF (Fig. 4a,b, Fig. S10). However, TIMP1 expression at both transcript and protein level was not significantly reduced in SP600125 pre-treated cells that were treated with EGF as compared to EGF treated vehicle control cells (Fig. 4c, Fig. S10). Further, inhibition of activation of MAPK8 led to significant reduction of EGF-mediated increase of MMP-9/TIMP1 ratio without affecting MMP-2/TIMP1 ratio (Fig. 4d). Additionally, effect of MAPK8 overexpression on HTR-8/SVneo cells invasion and MMP-2 and MMP-9 expression was also performed. MAPK8 overexpression led to significantly increased invasiveness of HTR-8/SVneo cells (Fig. S11). Further, MAPK8 overexpression significantly increased *FAS*, *MMP-2*, and *MMP-9* expression (as observed at transcript level) as compared to untreated control HTR-8/SVneo cells (Fig. S11). Whereas, MAPK8 overexpression led to significant reduction in *TIMP1* expression as compared to untreated control HTR-8/SVneo cells (Fig. S11).

### FAS silencing led to reduced HTR-8/SVneo cells invasion by inhibition of MMP-2, MMP-9 expression after EGF treatment

**Figure 5:**
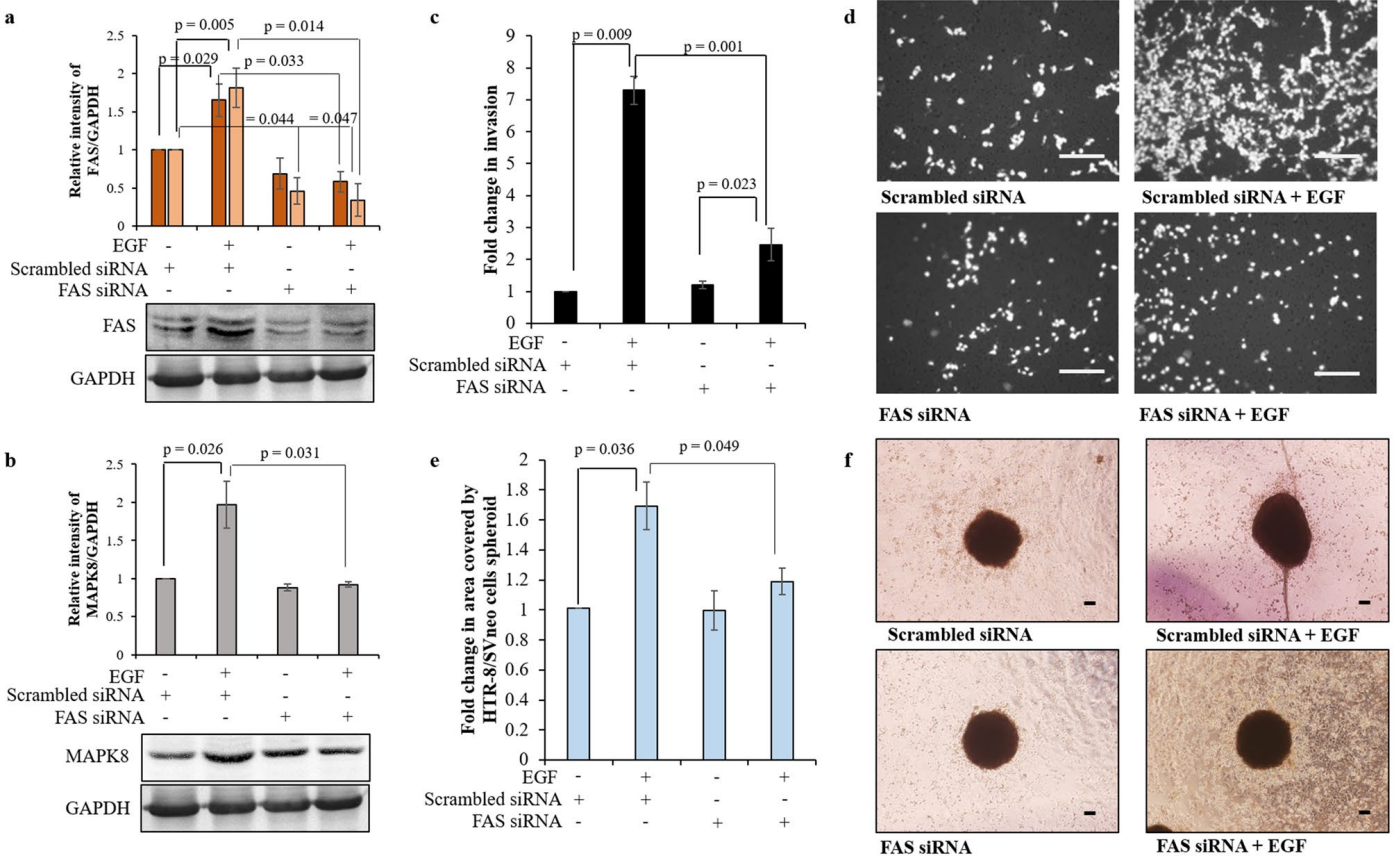
FAS silencing reduced EGF-mediated increased MAPK8 expression and HTR-8/SVneo cell invasion. Densitometric profile of FAS (**a**) and MAPK8 (**b**) with respect to GAPDH as loading control in FAS siRNA transfected HTR-8/SVneo cells treated with or without EGF as compared to scrambled siRNA transfected cells without EGF treatment. Representative cropped blots for FAS (**a**), MAPK8 (**b**) and GAPDH from one of the three independent experiments are appended below respective panels. Data in (**a**) and (**b**) are expressed as mean ± S.E.M of three independent experiments. Uncropped blots from three independent experiments are shown in Supplementary Fig. S12. **(c**) Fold change in invasion of FAS siRNA transfected HTR-8/SVneo cells subsequently treated with and without EGF as compared to scrambled siRNA transfected HTR-8/SVneo without EGF treatment. Data in (c) is means ± SEM of three independent experiments performed in duplicates. **(d**) Representative photographs of invading cells in various treatment groups on 0.8 µm pore size transwell membranes as observed under microscope after processing for invasion assay. Scale bars, 5 µm. (**e**) Fold change in the area covered by individual FAS siRNA transfected HTR-8/SVneo spheroids and control siRNA transfected HTR-8/SVneo spheroids, respectively, in the presence/absence of EGF after 24 h. Each bar represents the relative area of spreading after normalization with untreated scrambled siRNA-transfected HTR-8/SVneo spheroids. (**f**) Representative photographs of the spheroids are appended. Scale bar represents 20 μm. Values are expressed as mean ± SEM of three independent experiments.

**Figure 6:**
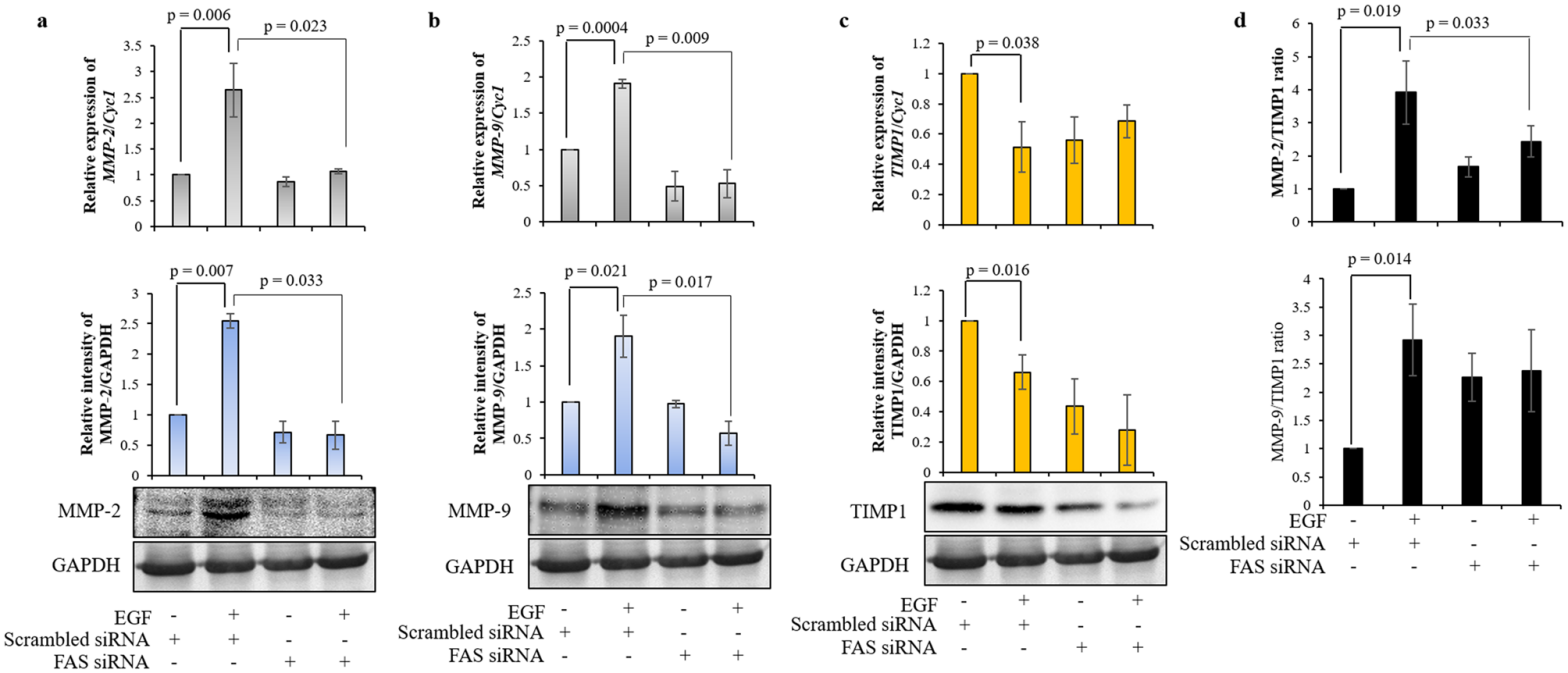
FAS silencing reduces EGF-mediated HTR-8/SVneo cell invasion by inhibiting activation of MMP-2 and MMP-9. Relative expression profiles at transcript and protein levels of MMP-2 (**a**), MMP-9 (**b**) and TIMP1 (**c**) with respect to *Cyc1* and GAPDH respectively as loading controls in FAS siRNA transfected HTR-8/SVneo cells treated with and without EGF as compared to scrambled siRNA transfected cells without EGF treatment. Representative cropped blots from one of the three independent experiments are appended below. The transcript data is shown as mean ± SEM of three independent experiments performed in triplicates and protein expression data is shown as mean ± SEM of three independent experiments. (**d**) Ratio of protein levels of MMP-2/TIMP1 and MMP-9/TIMP1. Uncropped blots from three independent experiments are shown in Supplementary Fig. S12.

To establish the role FAS during trophoblast invasion, FAS was knocked down using siRNA and silenced cells were used to study the effect on their invasion in presence or absence of EGF. Scrambled siRNA transfected HTR-8/SVneo cells were used as control group. FAS silencing significantly decreased EGF-mediated increased expression of FAS as compared to its observed expression in EGF treated scrambled siRNA transfected cells (Fig. 5a, Fig. S12). Concomitant significant decrease in MAPK8 activation was also observed in EGF treated FAS silenced cells as compared to scrambled siRNA transfected cells treated with EGF (Fig. 5b, Fig. S12). FAS silencing led to significantly reduced EGF-mediated increased HTR-8/SVneo cell invasiveness (Fig. 5c,d). However, no significant effect on HTR-8/SVneo cells basal invasiveness was observed after FAS silencing (Fig. 5c,d). Additionally, FAS silencing also significantly reduced EGF-mediated HTR-8/SVneo spheroid cells spreading in a co-culture experiment as compared to EGF treated siRNA control transfected cells (Fig. 5e,f). Transcript and protein levels of MMP-2 and MMP-9 in FAS silenced cells treated with EGF were significantly reduced when compared to EGF treated scrambled siRNA transfected control cells (Fig. 6a,b, Fig. S12). However, TIMP1 expression both at transcript and protein levels was not significantly reduced in EGF treated FAS silenced cells when compared to scrambled siRNA transfected cells treated with EGF (Fig. 6c, Fig. S12). Further, FAS silencing led to significant reduction of EGF-mediated increase of MMP-2/TIMP1 ratio without affecting MMP-9/TIMP1 ratio (Fig. 6d).

### Expression of miR-92a-1-5p in HTR-8/SVneo cells by EGF is regulated by activation of STAT1 and STAT3

**Figure 7:**
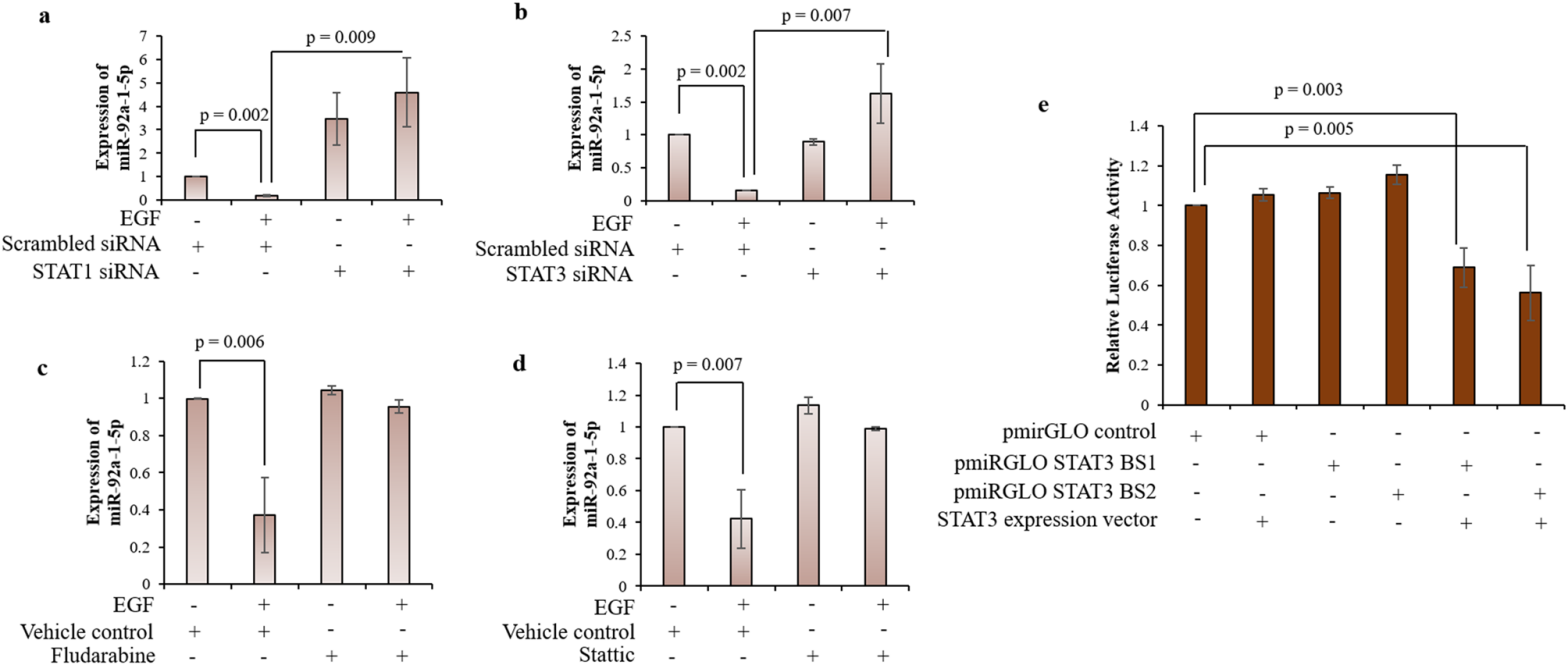
Effect of STAT1 and STAT3 silencing and inhibition by Fludarabine and Stattic in HTR-8/SVneo cells on the expression of miR-92a-1-5p in presence or absence of EGF. (**a**) Expression of miR-92a-1-5p by qRT-PCR in HTR-8/SVneo cells transfected with STAT1 siRNA treated with or without EGF for 24 h as compared to untreated scrambled control siRNA transfected cells. (**b**) Expression of miR-92a-1-5p by qRT-PCR in HTR-8/SVneo cells transfected with STAT3 siRNA treated with or without EGF for 24 h as compared to untreated scrambled control siRNA transfected cells. (**c**) Expression of miR-92a-1-5p by qRT-PCR in HTR-8/SVneo cells pre-treated with or without Fludarabine (4 h) and subsequently treated with or without EGF for 24 h. (**d**) Expression of miR-92a-1-5p by qRT-PCR in HTR-8/SVneo cells pre-treated with or without Stattic (1 h) and subsequently treated with or without EGF for 24 h. Each bar represents relative expression after normalization with the miR-191 used as an internal miRNA normalizer. Data in (**a**), (**b**), (**c**) and (**d**) is mean ± SEM of three independent experiments performed in triplicates. Synthetically synthesized STAT3 binding sites present in the promoter region of miR-92a-1-5p (BS1 corresponding to 492–510 nt and BS2 corresponding to 777–795 nt position from miRNA precursor encoding region, table S1 and S3) were cloned upstream of the PKG promoter of the firefly luciferase gene in pmirGLO Dual-Luciferase vector. (**e**) shows the relative luciferase activity determined from HEK-293T cells co-transfected with clones possessing the synthetically synthesized STAT3 binding sites in pmirGLO Dual-Luciferase vector with STAT3 mammalian expression vector, as indicated. The data are represented as the mean of three experiments ± S.E.M. performed in duplicates.

Reciprocal interactions of miRNAs and STATs are emerging to be important in regulating cancer-promoting inflammation, migration, malignant transformations and tumor cell metastasis^16^. Putative STAT1 and STAT3 binding sites in the promoter regions (5 kb upstream) of miR-92a-1-5p, using Genomatix MatInspector software were predicted (table S3)^17^. STAT1 and STAT3 are activated after EGF treatment of HTR-8/SVneo cells and play an important role in their increased invasion^18^. Additionally, STAT1 and STAT3 binding sites are present in the promoter region of miR-92a-1-5p. Hence, effect of STAT1 and STAT3 silencing and their inhibitors (Fludarabine for STAT1 and Stattic for STAT3) was investigated on the EGF-mediated expression levels of miR-92a-1-5p by qRT-PCR. miR-92a-1-5p showed increased basal level expression when STAT1 was silenced in HTR-8/SVneo cells, and EGF addition was not able to reduce the levels of miR-92a-1-5p in STAT1 silenced HTR-8/SVneo cells which otherwise was observed in EGF treated scrambled siRNA transfected HTR-8/SVneo cells (Fig. 7a). However, no increase in basal expression of miR-92a-1-5p was observed when STAT3 was silenced in HTR-8/SVneo cells when compared to untreated scrambled siRNA transfected cells (Fig. 7b). Treatment of STAT3 silenced cells with EGF failed to reduce the miR-92a-1-5p expression, which was observed in EGF treated scrambled siRNA transfected cells (Fig. 7b). Further, expression levels of miR-92a-1-5p in fludarabine or stattic pre-treated HTR-8/SVneo cells that were subsequently treated with EGF were not reduced as usually observed in EGF treated HTR-8/SVneo cells without any pre-treatment (Fig. 7c,d).

To confirm that the reduced expression of miR-92a-1-5p is due to direct regulatory relationship between STAT1/3 and miR-92a-1-5p, two synthetic oligos [Binding site 1 (BS1) and Binding site 2 (BS2)] corresponding to the promoter region of miR-92a-1-5p encompassing one STAT3 binding site each were made (table S1). These were cloned upstream of PKG promoter firefly luciferase gene in pmirGLO Dual-Luciferase expression vector. Co-transfection of STAT3 mammalian expression vector (which led to increased expression of total STAT3, Fig. S13) and pmirGLO plasmid with STAT3 binding sites pertaining to miR-92a-1-5p promoter significantly reduced firefly luciferase activity by 28% in case of BS1 and 39% in case of BS2 as compared to when cells were transfected with only pmirGLO plasmid (Fig. 7e). Further, no reduction in firefly luciferase activity was observed when empty pmirGLO was co-transfected with STAT3 mammalian expression vector (Fig. 7e).

## Discussion

Understanding the potential roles of the various miRNAs in trophoblast invasion, migration, and differentiation as regulatory factors can help in understanding the effect of their differential expression profiles observed in pregnancy complications. The association of placental pathological conditions and the placenta- and trophoblast-specific miRNA expression patterns have been described previously^19–21^. In the present study, the role of miR-92a-1-5p and miR-19a-5p during EGF-mediated HTR-8/SVneo cells invasion has been investigated. Both miR-92a-1-5p and miR-19a-5p belong to the miR-17-92 cluster of miRNAs and show reduced expression when HTR-8/SVneo cells are treated with EGF. MicroRNAs that belong to same clusters tend to be co-expressed/repressed, and can regulate overlapping sets of target genes^22^. Thus, the effect of overexpression of both miR-92a-1-5p and miR-19a-5p was investigated on EGF-mediated HTR-8/SVneo cell invasion. While overexpressing miR-19a-5p had no effect on EGF-mediated or basal HTR-8/SVneo cell invasion (Fig. S3); miR-92a-1-5p inhibition significantly up-regulated basal invasiveness of HTR-8/SVneo cells (Fig. S4), while its overexpression significantly reduced EGF-mediated HTR-8/SVneo cell invasion (Fig. 1). Role of increased expression of miR-92a-1-5p is observed in cervical cancer cells, and is believed to promote cell viability and invasion^14^. As opposed to the observed increased miR-92a-1-5p expression in cervical cancer cells, inhibition of miR-92a-1-5p was observed in HTR-8/SVneo trophoblast cells during increased invasion. These results point towards the significance of regulating miR-92a-1-5p expression during the process of invasion, as reduced miR-92a-1-5p expression might be responsible for controlled trophoblast invasion while its increased levels may be central during uncontrolled cancer cell invasion. The disparity in the regulation of cancer and trophoblast cell invasion, and BeWo cell differentiation^23^ by miR-92a-1-5p might be due to the differences in effectors molecules expressed during these processes in different cell types.

To identify the target proteins of miR-92a-1-5p during EGF-mediated HTR-8/SVneo cell invasion, bioinformatics tools were utilized to gather an idea about which genes out of the identified differentially expressed genes by NGS (Fig. S5) after EGF treatment showed putative binding with miR-92a-1-5p. *MAPK8*, *TCF7*, *CD151* and *FAS* were identified to be miR-92a-1-5p targets with binding sites with the help of TargetScan, miRTarBase and miRanda (miRNA target predicting softwares). The transcript levels of *TCF7* did not change after overexpression of miR-92a-1-5p in EGF treated HTR-8/SVneo cells, but significant decrease in transcripts of *CD151* was observed. The increased transcript levels of *CD151* after EGF treatment did not corroborate with its protein levels observed in EGF treated cells. The change in transcript level in EGF treated cells was only ~ twofold higher as compared to untreated cells. Thus, the increase in transcript may have not transcribed into protein expression, as often transcript and protein level do not correlate if the fold change observed at transcript level is low^24^. Transcript levels of *MAPK8* and *FAS* were observed to be altered by miR-92a-1-5p. miRNAs can mediate transcriptional gene activation or silencing, suggesting thereby that miRNAs may function not only at the post-transcriptional level^25^. The protein levels of MAPK8 and FAS in EGF treated cells with overexpressed miR-92a-1-5p corroborated with their transcript profile. In addition to controlling the transcript expression, there is a direct regulatory effect of miR-92a-1-5p expression observed on MAPK8 and FAS in Luciferase reporter gene assay, and confirmed by rescue of luciferase activity by mutants of their respective miRNA binding sites (Fig. S9). MAPK8/JNK1 is a member of the mitogen-activated protein kinases (MAPK) family of serine-threonine protein kinases that control cellular response to extracellular stimuli, and participate with various signaling pathways through cross-talks^26^. The down-regulation of MAPK8/JNK1 is reported to reduce human oral cancer cell migration by inhibiting MMP-9 enzymatic activity^27^. Additionally, MAPK8/JNK1 and its target transcription factor AP1 are also reported to mediate TGF-β1-induced epithelial to mesenchymal transition^28–30^; these reports support the observed reduced expression of MMP-2 and MMP-9 after MAPK8 inhibition and its role during EGF-mediated HTR-8/SVneo cells invasion.

**Figure 8:**
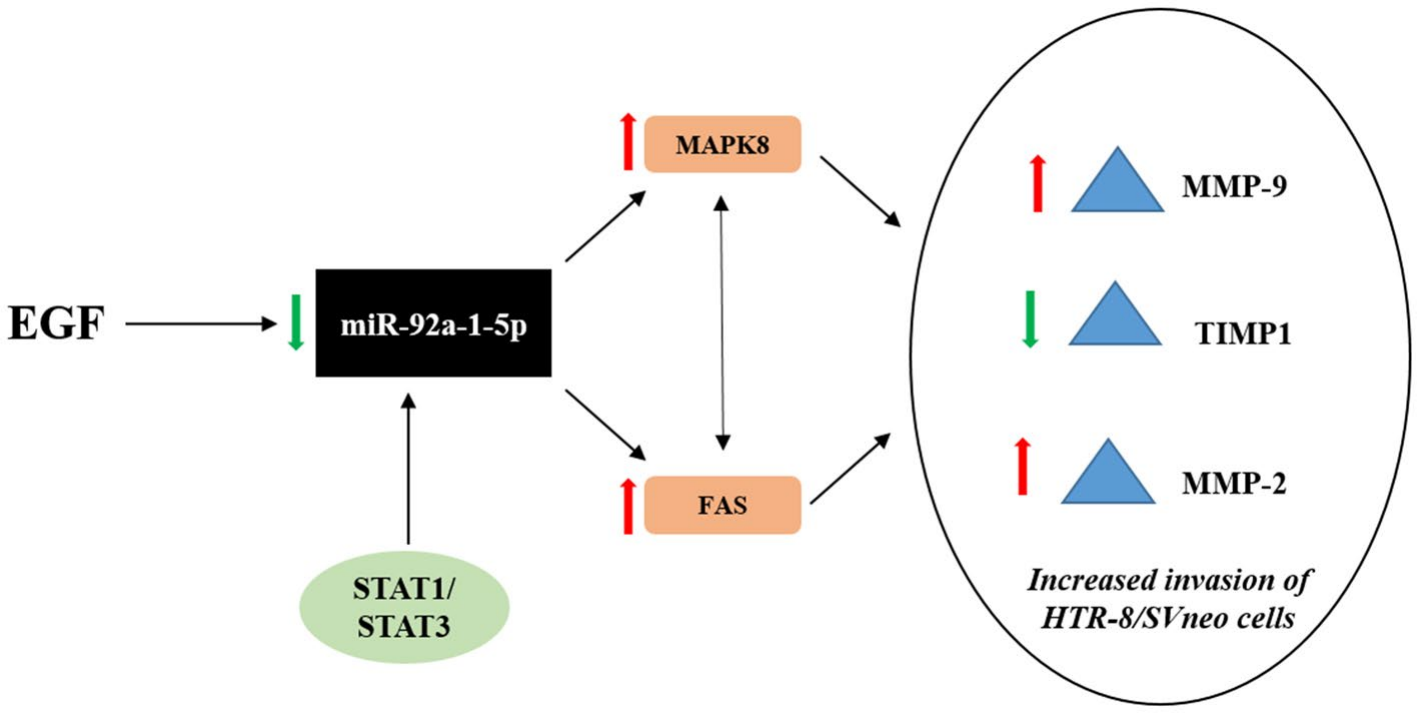
Graphical representation of the effect of reduced miR-92a-1-5p expression during EGF-mediated increased HTR-8/SVneo cell invasion. EGF leads to reduced expression of miR-92a-1-5p in HTR-8/SVneo trophoblastic cells; reduced miR-92a-1-5p leads to increased invasion of HTR-8/SVneo cells by targeting MAPK8 and FAS, which increases expression of MMP-2 & MMP-9, and decreases expression of TIMP1. EGF activated STAT1 and STAT3 may lead to reduced expression of miR-92a-1-5p by binding to the miRNA precursor encoding region as inhibition of STAT1 and STAT3 abrogates reduction of miR-92a-1-5p expression in EGF treated HTR-8/SVneo cells.

CD95/FAS is a type I transmembrane receptor which contains an intracellular death domain. CD95/CD95L system regulates apoptosis^31^, expression of pro-inflammatory cytokines and chemokines^32,33^, cell cycle progression^34^ and even cell migration & invasiveness^35^. Similar to what is observed in carcinoma MCF7 cells and ovarian cancer SK-OV-3 cells; reduction in EGF-mediated invasion was observed in FAS siRNA transfected HTR-8/SVneo cells. Further, loss of increased MAPK8, MMP-2 and MMP-9 expression was observed in EGF treated FAS silenced cells as compared to control siRNA transfected cells treated with EGF. Inhibition of MAPK8 or FAS, inhibited each other’s activation and reduced EGF-induced MMP-2 and MMP-9 expression suggesting that activating signals for the expression of these MMPs are transduced through MAPK-FAS cross-communication. It was observed that EGF-mediated HTR-8/SVneo cells invasion was completely inhibited by pre-treatment with SP600125 (MAPK8 inhibition), while FAS silencing reduced it by ~ 2.76 fold. This may be due to reduction in MMP-9/TIMP1 ratio after pre-treatment with SP600125 and MMP-2/TIMP1 but not MMP-9/TIMP1 ratio after FAS silencing respectively. From the above results it can be concluded that reduced expression of miR-92a-1-5p leads to loss of its repression control on MAPK8 and FAS when HTR-8/SVneo cells are treated with EGF (Fig. 8). Thus, increased expression of MAPK8 (Fig. S11) and FAS leads to increased MMP-2 and MMP-9, and reduced expression of TIMP-1 which increase the invasiveness of HTR-8/SVneo trophoblastic cells (Fig. 8).

Transcription factors (TFs) play a crucial role in controlling gene expression at the transcriptional and post-transcriptional levels. As many of the miRNAs lie in the introns/exons of other genes, therefore are regulated together with the gene. TFs thus also control miRNA expression in the cell. EGF treatment of HTR-8/SVneo cells leads to STAT1 and STAT3 activation^18^; and STAT1 and STAT3 in either homodimer form or heterodimer form act as transcription factors. As multiple binding sites for STAT1 and STAT3 were present in the 5 kb upstream promoter region of miR-92a-1-5p (table S3), effect of STAT1 & STAT3 silencing was determined on the expression of miR-92a-1-5p. Increased basal miR-92a-1-5p expression was observed in STAT1 silenced HTR-8/SVneo cells and treatment with EGF was not able to reduce the increased expression level of miR-92a-1-5p (Fig. 7a). This may be due to the loss of STAT1 repression of miR-92a-1-5p in its un-phosphorylated form, as STATs in their un-phosphorylated state are still able to alter gene expression and function as transcription factors^36^. This statement is further supported by the observed lack of decrease in expression of miR-92a-1-5p when only STAT1 phosphorylation is inhibited in the cells by using fludarabine (Fig. 7c). Further, transcription factors like AP-2 and p53 are reported to be up-regulated by EGF-mediated trophoblast cell invasion^37,38^; and multiple binding sites for both these transcription factor are also present in 5 kb upstream of the miR-92a-1-5p encoding region. It may be possible that in absence of STAT1 repression of miR-92a-1-5p, other transcription factors might be possibly altering miR-92a-1-5p expression during EGF-mediated HTR-8/SVneo cells invasion. Under the stimulation of cytokines, STAT1 is proven to directly bind to the promoter region of *C13orf25* and transcriptionally facilitate the expression of miR-17-92 cluster in keratinocytes^39^. Whereas, activated STAT3 binds to the promoter region of the miR-17-92 cluster and regulates its expression^11^. A direct regulatory effect of the two synthetically synthesized STAT3 binding sites was observed when co-transfection of STAT3 mammalian expression vector and pmirGLO plasmid with STAT3 binding site pertaining to miR-92a-1-5p promoter region was performed. Thus, failure to decrease the EGF-mediated miR-92a-1-5p expression after STAT1/3 silencing or inhibition may be due to the direct regulation of the downstream expression of miR-92a-1-5p. Further, the loss of EGF-mediated reduction in miR-92a-1-5p expressions in STAT1 as well as STAT3 silenced HTR-8/SVneo cells point towards possible heterodimer formation of phosphorylated STAT1 and STAT3. This is also supported by the *in-silico* predicted overlapping STAT1 and STAT3 binding sites (table S3), 5 kb upstream of miR-92a-1-5p precursor encoding region by Genomatrix MatInspector software.

TFs and miRNAs often coordinate and regulate shared target genes^40^, thus, forming a regulatory unit or a feed-forward loops (FFLs), in which miRNA can regulate TFs and target genes or TF can regulate miRNA and target genes, and both of them can co-regulate target genes^41^. Depending upon the master regulator, the FFLs (regulatory unit) can be classified into three groups: (1) miRNA-TF FFL, miRNA acts as a master regulator and regulates TFs and target genes expression; (2) TF-miRNA FFL, TF acts as a master regulator and regulates miRNA and target gene expression; and (3) TF = miRNA FFL, in which TF and miRNA mutually regulate each other’s expression as well as the target genes. On the basis of the results presented in this study, EGF-mediated invasion is mediated through TF-miRNA FFL, where STAT1/3 (TF) inhibit miR-92a-1-5p expression, and decrease in miR-92a-1-5p expression leads to increase in expression of MAPK8 and CD151 (Fig. 8).

This study has used HTR-8/SVneo cell line which is a transformed trophoblast cell line; thus, key experiments need to be performed in primary EVT cells or primary tissue sections to corroborate the findings published in the present study. Low levels of EGF are reported in preeclampsia^42^. Therefore, these results if, reproduced using primary EVT cells from women with preeclampsia will help in understanding the significance of reduced EGF and increased miR-92a-1-5p expression in preeclampsia.

## Methods

### Cell culture

The HTR-8/SVneo cells (SV40 large T antigen transformed first trimester human trophoblast cells) and Ishikawa cells (established from endometrial adenocarcinoma) were cultured in 10% heat-inactivated FBS enriched mixture of DMEM + Ham’s F-12 (1:1) medium additionally supplemented with an antibiotic and antimycotic cocktail (100 U⁄mL penicillin; 100 μg⁄mL streptomycin; 0.25 μg⁄mL amphotericin B) at 37 °C in humidified 5% CO_2_. At 70–80% confluency, the cells were passaged. Every third passage, HTR-8/SVneo cells were maintained in Geneticin (G418) sulphate (at 75 µg/mL)^43^. The HEK-293T cells (derived from HEK 293 cell line expressing a mutant version of the SV40 large T antigen, obtained from ATCC, VA, USA) were cultured in 10% heat-inactivated FBS enriched DMEM medium supplemented with the antibiotic and antimycotic cocktail at 37 °C in humidified 5% CO_2_.

### miRNA overexpression/inhibition using miRIDIAN miRNA Mimic/Inhibitor

Method of reverse transfection was employed for miRNA mimic/inhibitor transfection to the cells. Mimics of miR-92a-1-5p & miR-19a-5p, inhibitor of miR-92a-1-5p, and their respective negative controls (25 nM) were mixed with Opti-MEM® in individual tubes to make up 125 µL. In separate tubes, 6 µL of Lipofectamine RNAiMAX was mixed with Opti-MEM to make a total volume of 125 µL. Both the tubes were incubated for 5 min at room temperature following which they (each tube of OptiMEM with mimic/inhibitor/control mixed with OptiMEM with Lipofectamine) were mixed and incubated at room temperature for 10 min. This mix of mimic/inhibitor/controls with Lipofectamine was added in individual wells of 6-well plate, to which 0.25 × 10^6^ cells/well were seeded in 750 µL Opti-MEM medium. After 6 h of incubation at 37 °C under humidified conditions of 5% CO_2_, 1 mL of 20% FBS supplemented DMEM + Ham’s F-12 (1:1) medium was added to the wells. Next day, the medium was replaced with 1 mL of 10% FBS supplemented DMEM + Ham’s F-12 (1:1) medium and the plates were kept for 48 h. After 48 h, the cells were trypsinized and cells viability was accessed by Trypan Blue exclusion test. miRNA mimic, inhibitor and control mimic/inhibitor transfected cells were processed for Matrigel invasion assay.

### Invasion assay

Matrigel matrix (50 µl of 1 µg/mL, BD Biosciences, San Jose, CA, USA) was coated on 0.8 µm transwell insert membrane (Greiner Bio, Kremsműnster, Austria) and incubated overnight to solidify in a 24-well plate at 37 °C. Upper chamber of the transwell was seeded with 0.1 × 10^6^ cells in 150 µL of DMEM + Ham’s F-12 (1:1) medium containing 1% FBS with or without 10 ng/ml of EGF (Life Technologies, Carlsbad, CA, USA) treatment. The lower chamber was filled with 300 µL of DMEM + Ham’s F-12 (1:1) medium supplemented with 1% FBS with or without 10 ng/ml of EGF. The cells were allowed to invade for 24 h in 5% humidified CO_2_ at 37 °C. The upper chamber was aspirated to remove excess cells and matrigel, and the membrane was cleaned with a cotton swab moistened in PBS. The cells were fixed with chilled methanol on the lower side of the membrane at 4 °C for 5–10 min and stained with 0.2 µM Hoechst 33,342 at 37 °C for 5 min. The membranes were visualized under oil immersion in fluorescent phase contrast microscope ECLIPSE TE2000-E and total number of cells on the membrane were counted using Image pro-plus software (developed by Media Cybernetics, Rockville, MD, USA). The number of cells on untreated control membrane were taken as one, and by dividing number of cells on treated transwell insert membrane by the number of cells on untreated control transwell insert membrane, fold change in invasion was calculated.

### Co-culture of HTR-8/SVneo spheroids with Ishikawa cells

HTR-8/SVneo spheroids were prepared by hanging drop method as described previously^44,45^. Briefly, 3,000 miR-92a-1-5p mimic transfected/control mimic transfected/FAS siRNA transfected/control siRNA transfected HTR-8/SVneo cells in 20 μL DMEM + Ham’s F12 medium supplemented with 10% FBS in form of a drop were plated on the lid of a petri dish. The lid was inverted on the PBS-filled petri dish and incubated at 37 ºC with 5% CO_2_ and 70% relative humidity for 72 h. In one of the experimental setup, spheroids formed by naïve cells were pre-treated with and without SP600125 (MAPK8 inhibitor) for 1 h. Subsequently, spheroids were plated on the top of monolayer of Ishikawa cells in the presence or absence of EGF in serum starved DMEM + Ham’s F12 plain medium. Following 24 h of co-incubation, images were taken and the area of spread by cells from spheroid was measured by ImageJ software^46^.

### Treatment with SP600125, Fludarabine and Stattic

HTR-8/SVneo cells (0.1 × 10^6^/well) were seeded in 6-well plates and kept overnight in 5% humidified CO_2_ at 37 °C, following which they were serum starved for 4 h. Cells were kept in 50 µM Stattic for 1 h to inhibit STAT3 phosphorylation and 50 µM Fludarabine for 4 h to inhibit STAT1 phosphorylation as standardized previously^47^. Serum starved cells were kept in 25 µM SP600125 for 1 h to inhibit MAPK8 activation. Pre-treated and vehicle control cells were treated with or without EGF (10 ng/mL) and then used to perform Matrigel invasion assay as described above or qRT-PCR and Western Blot as described below.

### *In-silico* analysis

Target genes of miR-92a-1-5p were predicted using *in-silico* miRNA target prediction tools like TargetScan, microRNA.org, miRDB, miRNet, miRanda and miRTarBase. Target genes of miR-92a-1-5p were then compared against observed differentially expressed genes in EGF (24 h) treated HTR-8/SVneo cells. Simultaneously, to identify putative STAT1 and STAT3 binding sites, 5 kb upstream region of miR-92a-1-5p precursor was extracted^48^, and STAT1 and STAT3 binding sites were predicted using Genomatix MatInspector software^17^.

### Western blot

HTR-8/SVneo cells (0.1 × 10^6^/well) seeded in six well plates (previous day) were serum starved for 4 h and then treated with or without EGF (10 ng/mL) for 24 h. After aspirating the culture medium, cells were lysed in protease and phosphatase inhibitor cocktail (50 µL/well, Roche Diagnostics GmbH, Mannheim, Germany) supplemented RIPA buffer (20 mM Tris HCl, 10% glycerol, 0.2 mM EDTA, 0.137 M NaCl and 1% NP-40). Three freeze–thaw cycles in liquid nitrogen of the lysates were performed, following which the lysates were centrifuged at 12,000*g* for 10 min at 4 °C and the supernatant was collected. Protein estimation in each sample was done as per manufacturer’s instructions using BCA protein estimation kit (Thermo Scientific, Waltham, MA, USA). Each sample containing 40 µg of protein (equivalent volume) was denatured in 5X reducing sample buffer (250 mM Tris·HCl, pH 6.8, 10% SDS, 30% (v/v) glycerol, 2 M β-mercaptoethanol, 0.05% (w/v) Bromophenol Blue) at 95 °C for 5 min and resolved on 0.1% SDS-10% PAGE. Resolved samples were transferred to a nitro-cellulose membrane. The membrane was blocked with 5% BSA in Tris buffered saline (TBS, 50 mM Tris HCl, 150 mM NaCl) for 1 h at room temperature on a rocker shaker. After blocking, the membrane was washed in TBS and incubated with primary antibodies against MMP-2, TIMP1 (Cell Signaling Technologies, Danvers, MA, USA), MMP-9 (Thermo Scientific, Waltham, MA, USA), MAPK8, CD151, FAS (Cloud Clone Corp, Houston, TX, USA) and GAPDH (Abgenex, Bhubneshwar, Orrisa, India) diluted in TBS-T (0.1% Tween) with 1% BSA at recommended dilutions at 4 °C overnight. Incubation in primary antibodies was followed by three 10 min washings in TBST and incubation in secondary antibodies (anti-mouse HRPO for MMP-9 and GAPDH, or anti-rabbit HRPO for MMP-2, TIMP1, MAPK8, CD151 and FAS, Cell Signaling Technologies, Danvers, MA, USA) at 1:2000 dilution for 1 h at room temperature. Blots were developed by chemiluminescence HRP substrate (Merck Millipore, Burlington, MA, USA) following three 10 min washings in TBST. Pictures of the chemiluminescent blots were taken using FluorChem E System (ProteinSimple, San Jose, CA, USA). Individual protein expression levels were measured by quantifying intensities of specific bands corresponding to the proteins being analyzed using Image J software^46^.

### Gene silencing by siRNA

HTR-8/SVneo cells (0.1 × 10^6^/well) were seeded and incubated overnight in 6-well plates. Next day, cells were transfected with either control siRNA or siRNAs for STAT1/STAT3/FAS (Pool of 3 siRNAs; Santacruz Biotechnology, Santa Cruz, CA, USA) at their optimized concentrations (40 pmol for STAT-1 and control siRNA and 20 pmol for STAT-3, FAS and control siRNA) using Lipofectamine 3,000. Cells were washed with Opti-MEM® medium and 750 µL of fresh Opti-MEM® medium was added into each well. In an Eppendorf tube, optimized concentration of each siRNA was mixed with Opti-MEM to make a total volume of 125 µL. In a separate tube, 6 µL of Lipofectamine 3,000 was mixed with 119 µL Opti-MEM medium and incubated for 5 min at room temperature. Both the solutions were mixed and incubated for 20 min at room temperature. The mixed solution was added dropwise in respective wells. After 6 h of incubation under humidified conditions of 5% CO_2_ at 37 °C, complete medium was added to the cells. After 48 h of transfection, cells were processed for various experiments.

### Gene overexpression using mammalian expression vector

In addition, HTR-8/SVneo cells were transfected with mammalian MAPK8 expression vector (100 ng), STAT3 expression vector (310 ng) and vector control using OptiMEM and Lipofectamine as described above. After 48 h of transfection, cells were processed for various experiments.

### Complementary DNA (cDNA) preparation for mRNA expression analysis

A mixture (M1) of dNTP (0.5 mM), oligo (dT) (50 pmol), random hexamer primers (50 pmol) and isolated total RNA (5 µg) was made and incubated for 5 min at 65 °C to open any secondary structures formed in the RNA. M1 was briefly chilled on ice, and to each sample a mixture (M2) of RT buffer (1X), RNase inhibitor Ribolock (20 U) and Maxima reverse transcriptase (200 U) was added. The cDNA was formed by incubation the mix (M1 + M2) at 25 °C for 10 min followed by 30 min at 50 °C. The reaction was terminated by heating at 85 °C for 5 min.

### cDNA preparation for miRNA analysis

RNA (100 ng) was used to prepare cDNA in a final volume of 10 µL. The reaction mixture contained ATP (0.1 mM), RTprimer (1 μM, 5‟-CAGGTCCAGTTTTTTTTTTTTTTTVN; Sigma Aldrich Inc.), deoxynucleotides (0.1 mM of each dATP, dCTP, dGTP and dTTP), poly(A) polymerase buffer (1 µL of 10X), poly(A) polymerase (1 unit) and MuLV reverse transcriptase (100 units). The reaction mixture was incubated for 1 h at 42 °C followed by 95 °C for 5 min for cDNA synthesis.

### Quantitative real time polymerase chain reaction (qRT-PCR)

The qRT-PCR reactions were performed in triplicates in 10 µL reactions to analyze the transcripts for *MAPK8*, *TCF7*, *CD151*, *FAS, MMP-1, MMP-2, MMP-3, MMP-7, MMP-9, MMP-12, MMP-15, MMP-16, MMP-21, MMP-23b, TIMP1, TIMP2, TIMP3,* & *TIMP4*; and miRNA’s miR-92a-1-5p & miR-19a-5p (Primer sequence for all mRNA and miRNA are given in table S4). The reaction mixture contained Maxima™ SYBR green master mix (1X), cDNA template (diluted 5 times for mRNA and 3 times for miRNA analysis) and forward & reverse gene specific primers (0.1 µM each). Quantitative polymerase chain reaction was performed in Stratagene Mx3005P (Agilent Technologies Inc., Santa Clara, CA, USA). Targeted sequence amplification was performed as follows: 10 min initial denaturation at 95 °C, followed by 40 amplification cycles of 15 s at 95 °C and 60 s at primer specific annealing temperature. Lastly, a dissociation curve analysis was carried out for 20 min at a temperature range of 60 to 95 °C. Gene-specific amplification was confirmed if a single peak was observed in the dissociation curve analysis. *Cyc1* and miR-191 were run in parallel to mRNA and miRNA samples respectively to normalize average threshold cycle (Ct) values. Results were calculated using ΔΔCt method, to determine the fold change in expression (relative expression) between the experimental and control groups.

### Luciferase reporter assay

Sense and antisense sequences corresponding to a 53-bp fragment from the 3′-UTR of MAPK8 and FAS with the respective predicted binding and mutated sites (MAPK8 position 2,237–2,243 and FAS position 2,480–2,488) were chemically synthesized (table S1, Eurofins Genomics). Nucleotide sequence corresponding to *Xho*I restriction site in the sense oligo and *Sal*I restriction site in the antisense oligo were appended at 5′ end for creating sticky ends upon annealing as described elsewhere^49^. Similarly, two different sense and antisense sequences [Binding site 1 (BS1, table S1) 492–510 nt and binding site 2 (BS2, table S1) 777–795 nt position from miRNA precursor encoding region, table S3] corresponding to 63-bp fragment from the promoter region upstream of miR-92a-1-5p encompassing the predicted STAT3 binding sites respectively were chemically synthesized (Eurofins Genomics). Nucleotide sequence corresponding to *Mlu*I restriction site in the sense oligo and *Bgl*II restriction site in the antisense oligo were appended at 5′ end for creating sticky ends upon annealing as described elsewhere^49^. The synthesized oligo’s were resuspended in 1X annealing buffer (10X annealing buffer: 100 mM Tris–HCl pH -7.5, 1 M NaCl, 10 mM EDTA) to make a final concentration of 100 µM. Sense and antisense (1 µL each) were diluted to 10 µM concentration in 1X annealing buffer and annealed by incubating at 95 °C for 6 min and 25 °C for 30 min. The annealed oligos were diluted twice, to make the final concentration of the oligo’s to 5 nM in double distilled water.

To construct luciferase reporter plasmids for FAS and MAPK8, the annealed synthetic oligos were cloned individually downstream to the firefly luciferase into *Xho*I-*Sal*I double digested pmirGLO Dual-Luciferase miRNA target expression vector (Promega, WI, USA). Similarly, to construct luciferase reporter plasmids for STAT3 binding sites, the annealed synthetic oligos were cloned individually upstream to the firefly luciferase into *Mlu*I-*Bgl*II double digested pmirGLO Dual-Luciferase miRNA target expression vector (Promega, WI, USA). For the luciferase reporter assay, HEK-293T cells (ATCC) were co-transfected with 250 ng of luciferase reporter plasmid harboring the wild type/mutant binding sites of MAPK8 and FAS respectively along with 25 nM mimic control/miR-92a-1-5p mimic using Lipofectamine RNAiMAX reagent in OptiMEM. Alternately, HEK-293T cells were co-transfected with 250 ng of luciferase reporter plasmid harboring the STAT3 binding site along with 310 ng of STAT3 mammalian expression vector (OriGene Technologies, MD, USA) using Lipofectamine RNAiMAX reagent in OptiMEM. After 48 h of transfection, cells were washed in PBS and lysed in Reporter lysis buffer (Promega), and luciferase activity was measured in a Sirius Tube Luminometer (Berthold Detection Systems, Bad Wildbad, Germany) using the Dual-Luciferase reporter assay kit (Promega) according to the manufacturer's instructions. Firefly luciferase activity was normalized to *Renilla* luciferase activity, and relative luciferase activity was calculated taking firefly luciferase activity of empty pmirGLO transfected cells as 100 percent.

### Statistical analysis

The results are expressed as mean ± SEM of three independent experiments as indicated in the figure legend. Statistical analysis was performed by comparing the means of the control and experimental sets by using one way ANOVA. Post-hoc Bonferroni correction was further applied to qRT-PCR experiments when more than two groups were analyzed for statistical significance. Individual *p* values are indicated in the figures. A value of *p* < 0.05 was considered to be statistically significant.

## Supplementary information

Supplementary Information.

## Data Availability

The data discussed in this publication has been deposited in NCBI's Gene Expression Omnibus ^50^ and are accessible through GEO Series accession number GSE124587 (https://www.ncbi.nlm.nih.gov/geo/query/acc.cgi?acc=GSE124587).
