# Supplementary material for: EGF-mediated reduced miR-92a-1-5p controls HTR-8/SVneo cell invasion through activation of MAPK8 and FAS which in turn increase MMP-2/-9 expression
Source: Sci Rep. 2020 Jul 23;10:12274. doi: 10.1038/s41598-020-68966-4 (PMC7378053; doi:10.1038/s41598-020-68966-4)
Supplement: Supplementary file 1 — Supplementary Information. [file 41598_2020_68966_MOESM1_ESM.pdf]

EGF-mediated reduced miR-92a-1-5p controls HTR-8/SVneo cell invasion through activation of MAPK8 and FAS which in turn increase MMP-2/-9 expression

Ankita Malik[1], Rahul Pal[2] and Satish Kumar Gupta[1]*

*Corresponding Author

Address for Correspondence

Dr. Satish Kumar Gupta, PhD
Emeritus Scientist
J. C. Bose National Fellow
Reproductive Cell Biology Lab
National Institute of Immunology
Aruna Asaf Ali Marg
New Delhi- 110 067
Phone: 091 11 26741249
Fax : 091 11 26742125
E-mail: skgupta@nii.ac.in
ORCID: 0000-0003-3717-0436

Supplementary Materials and Methods

miRNA next generation sequencing (NGS)

HTR-8/SVneo cells (0.1 X 10^6/well) were seeded in 6-well culture plates and were allowed to grow overnight at 37°C in humidified atmosphere of 5% CO_2. Next day, the cells were subjected to FBS starvation for 4 h followed by treatment with EGF (10 ng/mL) in DMEM + Ham's F-12 (1:1) medium without FBS for 24 h at 37°C in humidified atmosphere of 5% CO_2. After 24 h, the supernatant was discarded and cells were lysed in Tri reagent for RNA isolation. Total RNA was isolated and the samples were outsourced to Next Generation Sequencing Lab, National Institute of Immunology, New Delhi, India. The small RNAs libraries were constructed using Truseq Small RNA sample preparation kit according to the manufacturer's instructions (Illumina, Inc., USA). Briefly, the total RNA (2 µg) was ligated with 3' adapter using T4 RNA ligase 2 truncated (NEB) and a 5' adapter using T4 RNA ligase 2. The ligation products were reverse transcribed using Superscript II Reverse Transcriptase and amplified with PCR (12 cycle). The PCR product was purified by electrophoresis using 6% Novex TBE PAGE Gel. The purified cDNA library for each sample was analyzed on Agilent 2100 Bioanalyzer using Agilent DNA high sensitivity kit. Deep sequencing of cDNA libraries were performed with the help of Illumina Genome analyzer II_X for 36 cycles.

A total of 2.8 GB of raw sequence data, comprising of untreated control and 24 h EGF treated samples, was imported into the CLC Genomics Workbench 6.5.1. The sequence reads were trimmed for adapter sequences and low quality base. The trimmed raw sequences were subjected to miRNA-sequence analysis, by mapping them to miRBase release 21 accounting for a maximum of two gaps or mismatches in each sequence. Unpaired group comparisons, based on Transcript Per Million (TPM), were chosen as expression values for comparison. Kal's Z test statistical analyses, based on p value < 0.05, fold change $\pm$ 1.5 and read count > 10 were used to filter the differentially expressed miRNA.

mRNA NGS

HTR-8/SVneo cells (0.1 X 10^6/well) were seeded in 6-well culture plates and were allowed to grow overnight at 37°C in humidified atmosphere of 5% CO_2. Next day, the cells were subjected to FBS starvation for 4 h followed by treatment with EGF (10 ng/mL) in DMEM + Ham's F-12

(1:1) medium without FBS for 24 h at 37°C in humidified atmosphere of 5% CO_2. After 24 h, the supernatant was discarded and cells were lysed in Tri reagent for RNA isolation. Total RNA was isolated and the samples were outsourced to Bionivid Technology Pvt Ltd. (Bangalore, India) for mRNA NGS. The RNA samples were first subjected to quality check (QC) using Bioanalyzer, RNA integrity number (RIN) for all samples was above 8. RNA above RIN value 5.0 is considered suitable for mRNA sequencing experiment. Thereafter, samples were processed for mRNA NGS employing *Illumina HiSeq system*. The raw data was collected as *fastq.gz* files. These files were used for data normalization and calculation of p-value to find statistically relevant differentially expressed genes. Normalized data was imported into GSTACK - Genomics cloud software.

Rescue Assay

For the rescue assay, HEK-293T cells (ATCC) were co-transfected with 250 ng each of luciferase reporter plasmid harboring the wild type and the mutated binding sites of MAPK8 & FAS respectively along with 25 nM mimic control/miR-92a-1-5p mimic using Lipofectamine RNAiMAX reagent in OptiMEM. After 48 h of transfection, cells were washed in PBS and lysed in Reporter lysis buffer (Promega), and luciferase activity was measured in a Sirius Tube Luminometer (Berthold Detection Systems, Bad Wildbad, Germany) using the Dual-Luciferase reporter assay kit (Promega) according to the manufacturer's instructions. Firefly luciferase activity was normalized to *Renilla* luciferase activity, and relative luciferase activity was calculated taking firefly luciferase activity of empty pmirGLO transfected cells as 100 percent.

miR-92a-1 – Stem loop precursor sequence

```
   ---cu   uac            c   u           uu
5'      uuc   acagguugggau ggu gcaaugcugug  u
        |||   |||||||||||| ||| |||||||||||
3'      gag   uguccggcccug uca cguuaugguau  c
   gguuu   --u            u   -           gu
```

Gives rise to two mature sequences namely -

miR-92a-1-5p – Previous ID: miR-92a-1*

AGGUUGGGAUCGGUUGCAAUGCU

miR-92a – Current ID: miR-92a-3p

UAUUGCACUUGUCCCGGCCUGU

Fig. S1 – miR-92a-1 stem loop precursor and mature sequences.

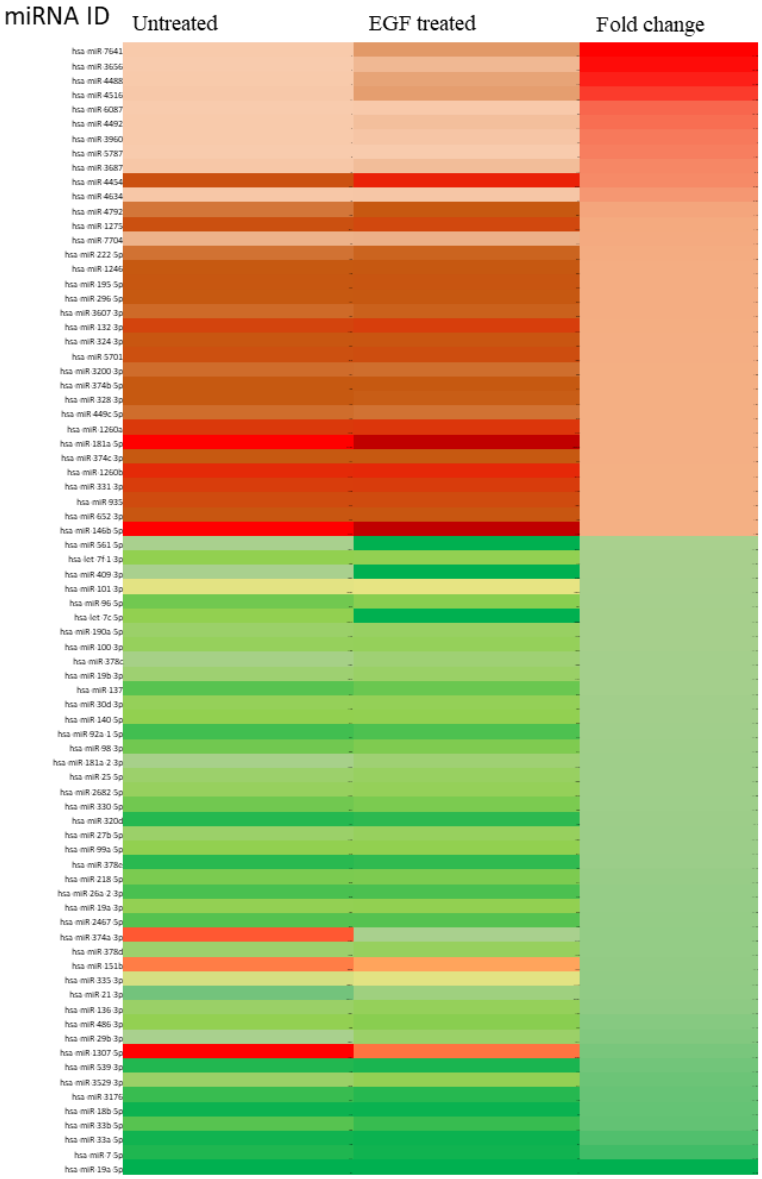

Fig. S2 – EGF-mediated differential expression of various miRNAs in HTR-8/SVneo cells. The data represented as heat map in this figure has been deposited in NCBI's Gene Expression Omnibus [Edgar, R., Domrachev, M., Lash, A. E. Gene Expression Omnibus: NCBI gene expression and hybridization array data repository. Nucleic Acids Res. **30,** 207-210 (2002)] and is accessible through GEO Series accession number **GSE124585** (**https://www.ncbi.nlm.nih.gov/geo/query/acc.cgi?acc=GSE124585**)

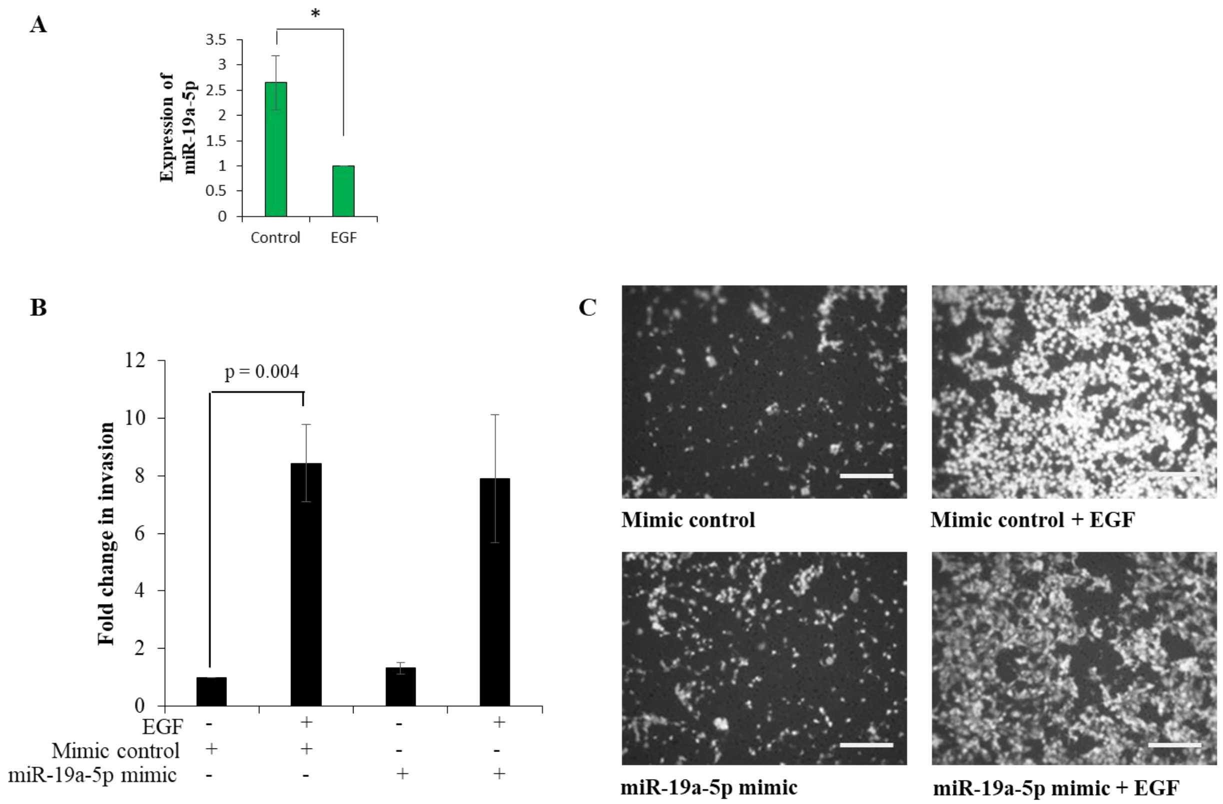

Fig. S3 - Reduced expression of miR-19a-5p in EGF treated HTR-8/SVneo cells and role of its mimic in invasion. HTR-8/SVneo cells were treated with EGF (10 ng/mL) for 24 h along with untreated control cells and used to perform qRT-PCR. Panel A shows relative expression of miR-19a-5p in EGF treated as compared to untreated HTR-8/SVneo cells. Values are expressed as mean ± SEM of three independent experiments performed in triplicates. HTR-8/SVneo cells transfected with miR-19a-5p mimic or control mimic were used to perform Matrigel invasion assay in presence or absence of EGF treatment for 24 h. Panel B shows fold change in invasion of EGF treated and untreated miR-19a-5p mimic transfected HTR-8/SVneo cells as compared to EGF untreated control mimic transfected HTR-8/SVneo cells. Values are expressed as mean ± SEM of three independent experiments performed in duplicates. Panel C shows representative photographs of invading cells in various treatment groups on 0.8 µm pore size transwell membranes as observed under microscope after processing for invasion assay. Scale bar represents 5 µm.

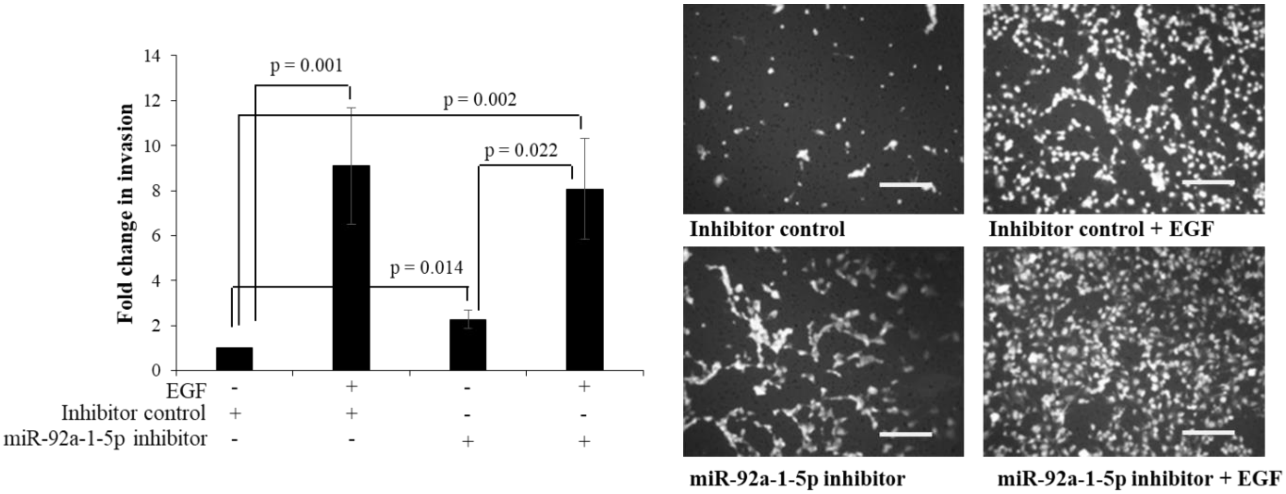

Fig S4 – Effect of EGF treatment on HTR-8/SVneo cells invasion in presence/absence of miR-92a-1-5p inhibitor. HTR-8/SVneo cells transfected with miR-92a-1-5p inhibitor or inhibitor control were used to perform Matrigel invasion assay in presence or absence of EGF treatment for 24 h. Bar graph shows fold change in invasion of the HTR-8/SVneo cells after treatment with EGF in inhibitor control cells and those treated with miR-92a-1-5p inhibitor followed by treatment with and without EGF as compared to cells that were neither treated with EGF nor miR-92a-1-5p inhibitor. Values are expressed as mean ± SEM of three independent experiments performed in duplicates. Representative photographs of invading cells in various treatment groups on 0.8 µm pore size transwell membranes as observed under microscope after processing for invasion assay are appended alongside. Scale bar represents 5 µm.

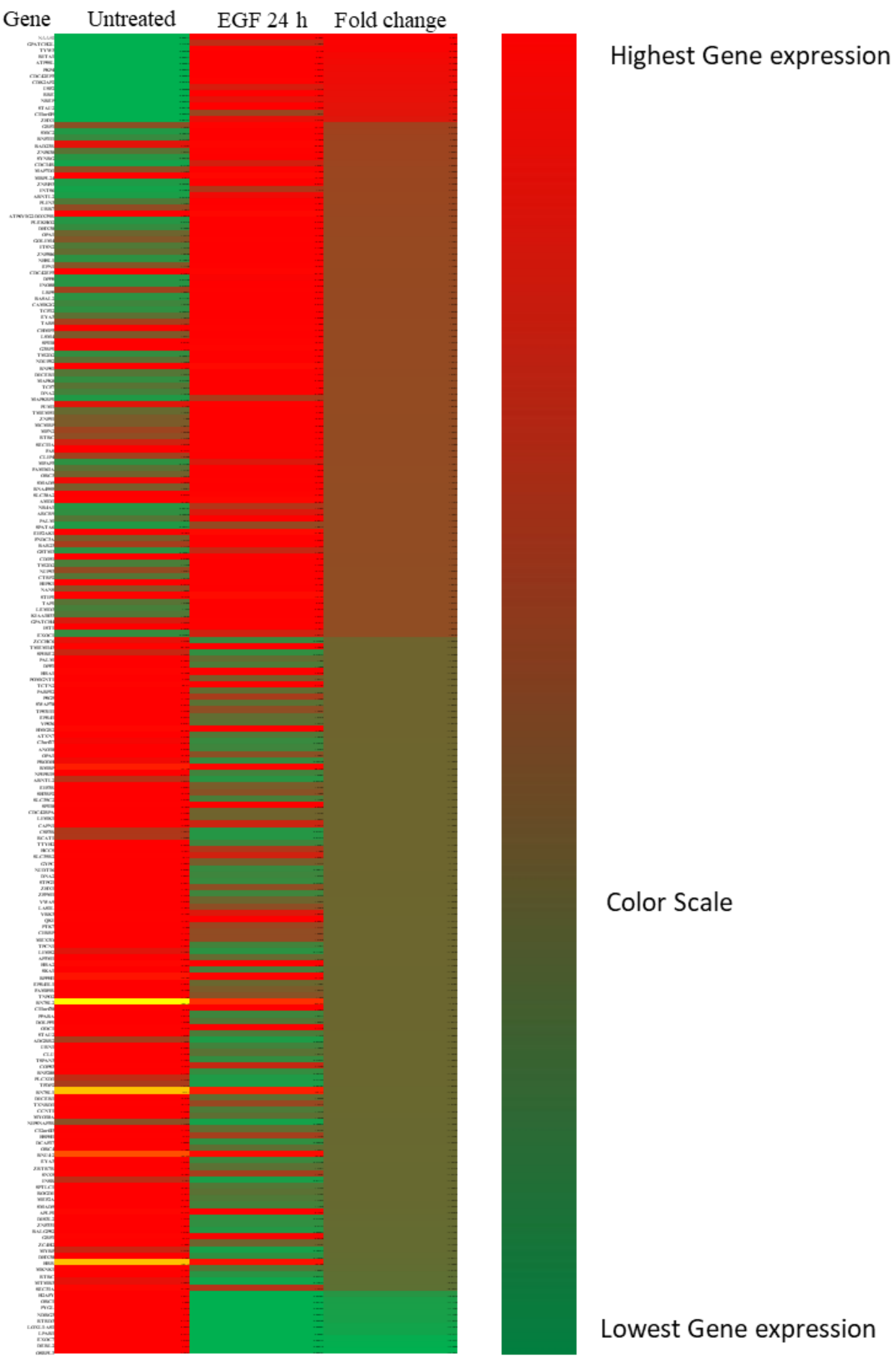

Fig. S5– Differentially expressed mRNA after EGF treatment of HTR-8/SVneo cells. The data represented as heatmap in this figure has been deposited in NCBI's Gene Expression Omnibus [Edgar, R., Domrachev, M., Lash, A. E. Gene Expression Omnibus: NCBI gene expression and hybridization array data repository. Nucleic Acids Res. **30,** 207-210 (2002)] and is accessible through GEO Series accession number **[GSE1245856](https://www.ncbi.nlm.nih.gov/geo/query/acc.cgi?acc=GSE1245856)** (**https://www.ncbi.nlm.nih.gov/geo/query/acc.cgi?acc=GSE1245856**)

MAPK8 – one site (TargetScan -7mer-A1 site)

Position (2237-2243) **5’ ....GCCAGCUGCCACUUGCCAACCAU…**
| | | | | |
miR-92a-1-5p **3’ UCGUAACGUUGGCUAGGGUUGGA**

TCF7– one site (TargetScan - 7mer-m8 site)

Position 614-620 **5' ...CACUGCUCUCAGCCUCCCAACCC...**
| | | | | | |
miR-92a-1-5p **3' UCGUAACGUUGGCUAGGGUUGGA**

CD151– two sites (TargetScan - 7mer-m8 sites)

Position 117-123 **5' ...AUAACCUCUGGGGACCCCAACCU...**
| | | | | | |
miR-92a-1-5p **3' UCGUAACGUUGGCUAGGGUUGGA**

Position 430-436 **5' ...UGGGCCUCUGCCCCUCCCAACCC...**
| | | | | | |
miR-92a-1-5p **3' UCGUAACGUUGGCUAGGGUUGGA**

FAS – three sites (miRanda, miRTarBase -7mer-A1 sites)

Position 1115 – 1133 **5’ …CCTAATGC – ACC - - - CCCAAACA…**
| | | | | | | | | | | |
miR-92a-1-5p **3’ UCGUAACGUUGGCUAGGGUUGGA**

Position 2063 – 2088 **5’ …TCTATT - TAACCCATGAGTCCCAAAGT…**
| | | | | | | | | | | | | |
miR-92a-1-5p **3' UCGUAACGUUGG - - - CU – AGGGUUGG**

Position (2460 – 2488) **5’ … CTCATGGCTTCACCTAGTGGCCCCAAGCA**
| | | | | | | | | | | | | | |
miR-92a-1-5p 3’ **UCGUAACG - - UUGG - - - - CUAGGGUUGGA**

Fig. S6 – Predicted consequential pairings of miR-92a-1-5p with its target genes in HTR-8/SVneo cells. Targets of miR-92a-1-5p miRNA were identified from observed differentially expressed mRNAs in EGF treated HTR-8/SVneo cells by *in-silico* analysis from TargetScan, miRanda and miRTarBase. The figure shows the predicted pairings of miR-92a-1-5p (blue) with its target genes (red) in HTR-8/SVneo cells at their 3’ UTR.

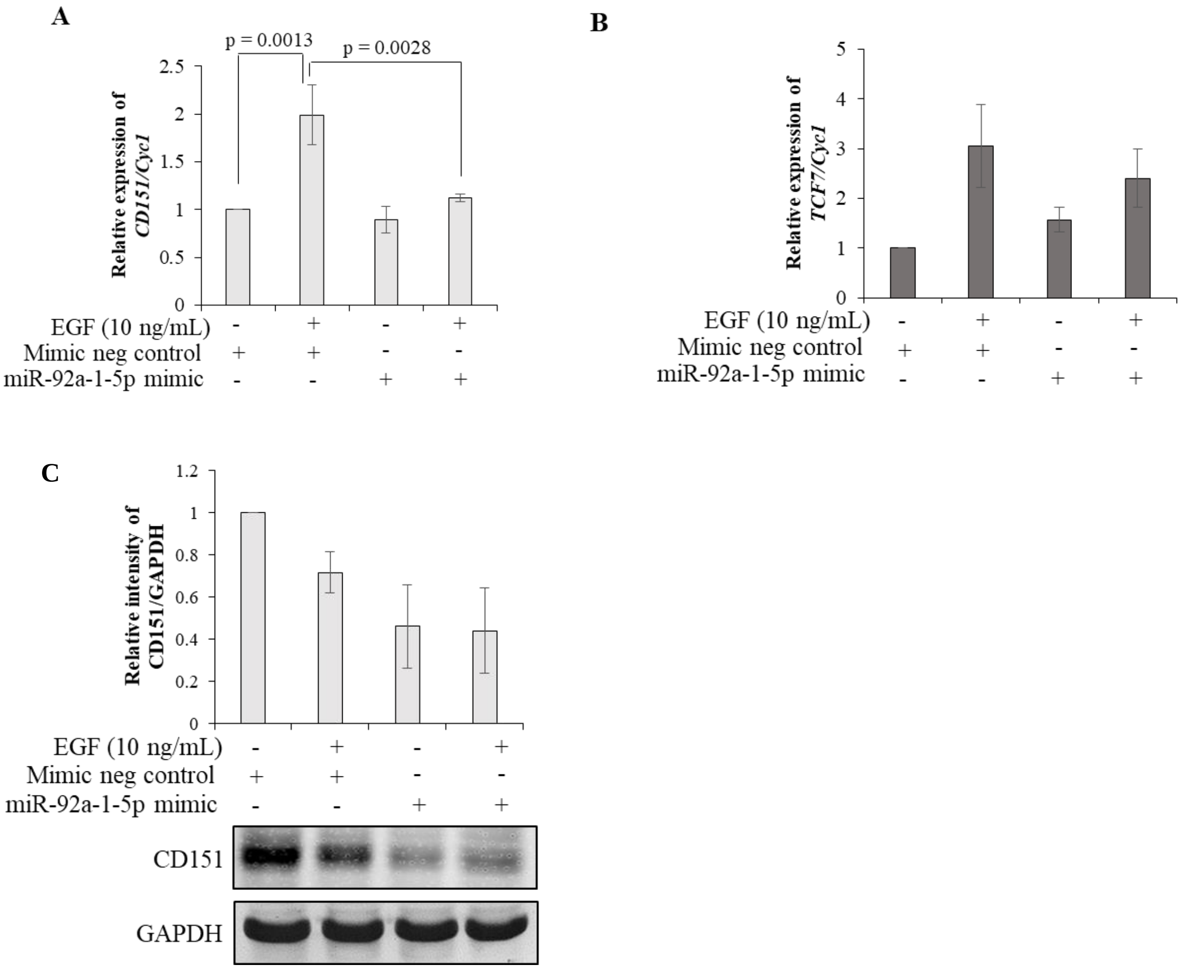

Fig. S7 - Effect of miR-92a-1-5p mimic on activation of CD151 and TCF7 in EGF treated HTR-8/SVneo cells. Relative expression of *CD151* (A) and *TCF7* (B) taking *Cyc1* as internal control in miR-92a-1-5p mimic transfected HTR-8/SVneo cells with or without treatment with EGF as compared to control mimic transfected cells without EGF treatment. Data is represented as mean ± SEM of three independent experiments performed in triplicates. (C) Densitometric profile of CD151 with respect to GAPDH as loading control from cell lysates of miR-92a-1-5p mimic transfected HTR-8/SVneo cells treated with or without EGF for 24 h as compared to control mimic transfected cells without EGF treatment. Representative blots from one of the three independent experiments are appended below. The values are expressed as mean ± SEM of three independent experiments.

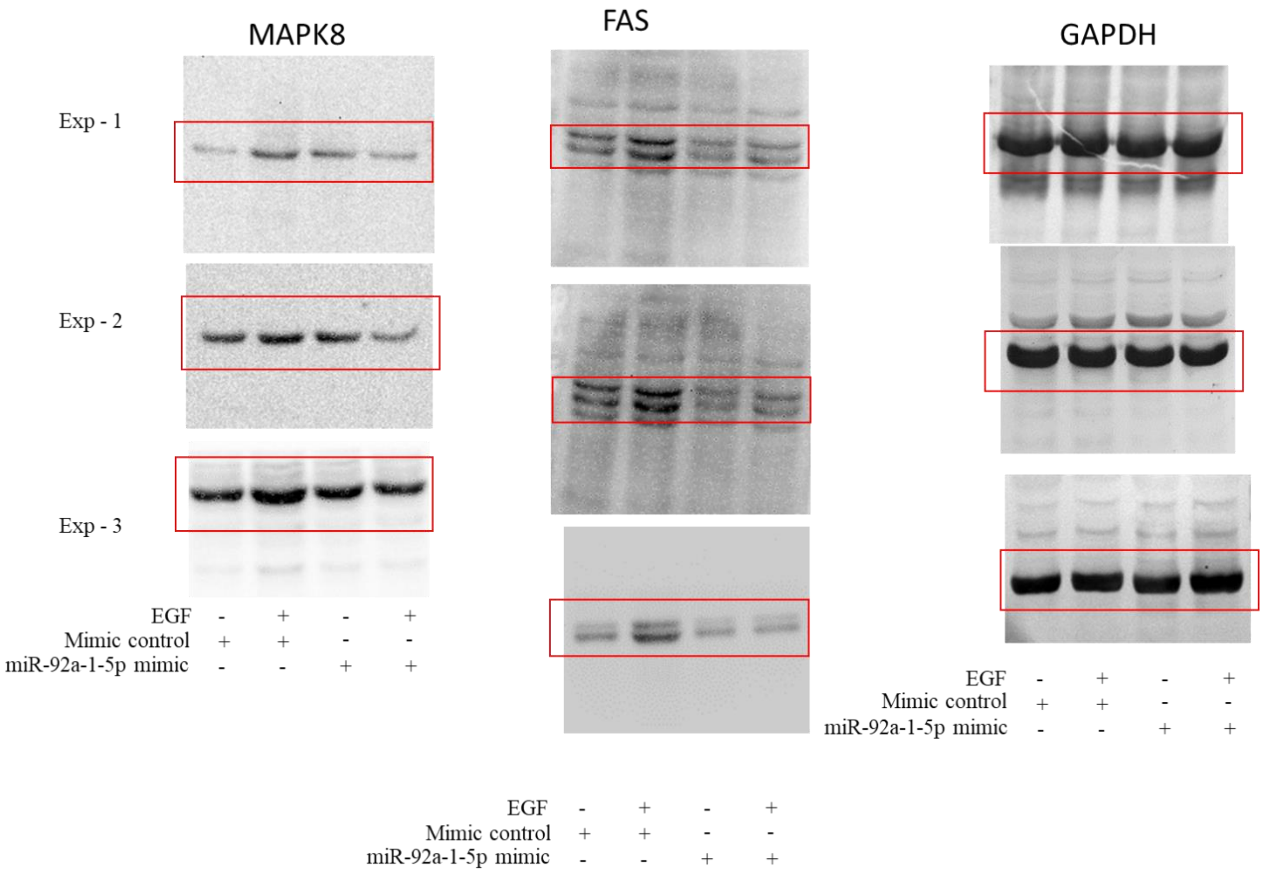

Fig. S8 - Effect of miR-92a-1-5p mimic on activation of MAPK8 and FAS in EGF treated HTR-8/SVneo cells. Cell lysates were prepared from HTR-8/SVneo cells transfected with 25 nM miR-92a-1-5p mimic, and mimic control with or without EGF treatment and Western blotting performed for determining MAPK8, FAS and GAPDH protein expression as described in *Methods*. Blots from three independent experiments are shown.

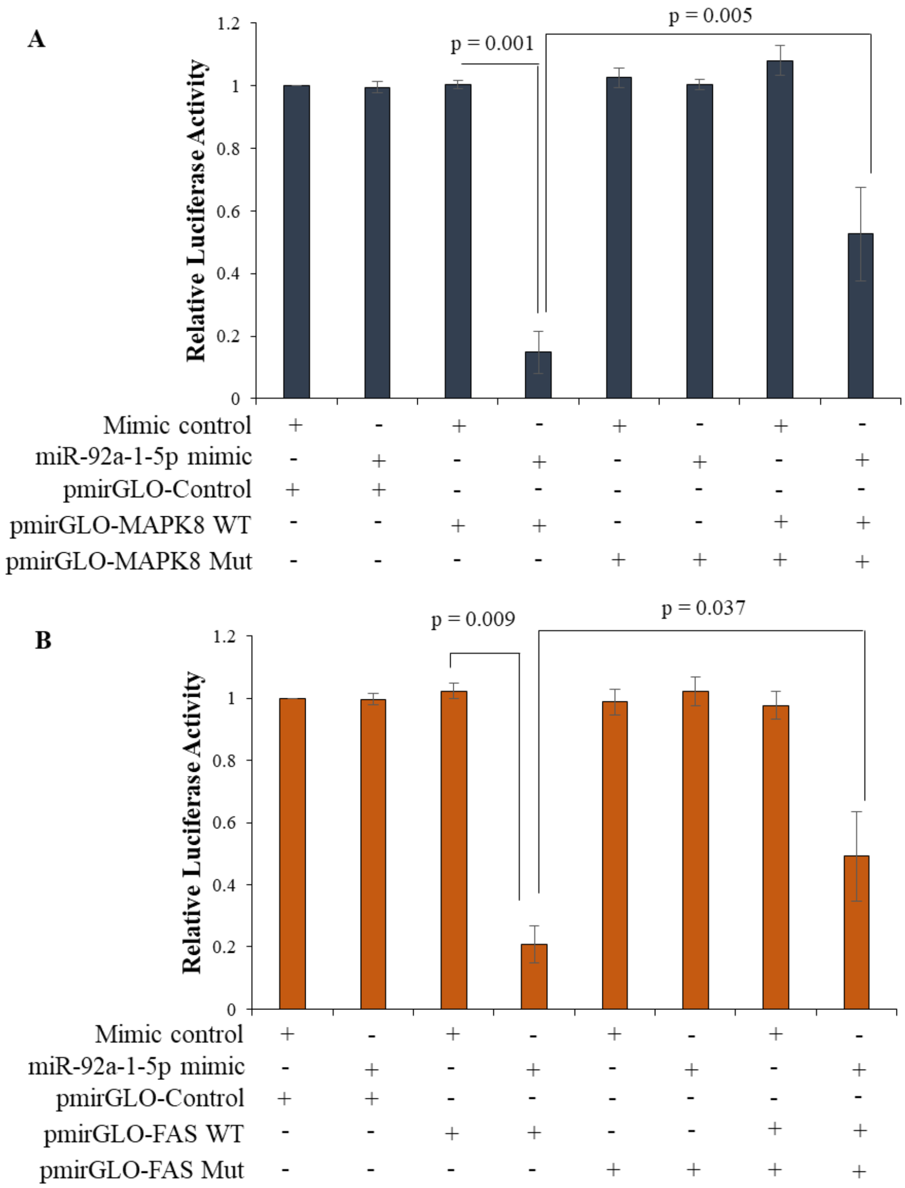

Fig. S9 – Rescue of luciferase activity by MAPK8/FAS Mut clone in presence of miR-92a-1-5p mimic and MAPK8/FAS WT clone. Wild type (WT) as well as mutated (Mut) MAPK8 and FAS complementary binding sites corresponding to miR-92a-1-5p seed sequence were synthesized and cloned downstream of the firefly luciferase gene under the control of the PGK promoter in pmirGLO Dual-Luciferase vector. (A) and (B) show the relative luciferase activity determined from HEK-293T cells co-transfected with clones possessing the WT and mutant binding site for miR-92a-1-5p corresponding to MAPK8 and FAS respectively in pmirGLO Dual-Luciferase vector with control or miR-92a-1-5p mimic, as indicated. The data are represented as the mean of four experiments ± S.E.M. performed in duplicates.

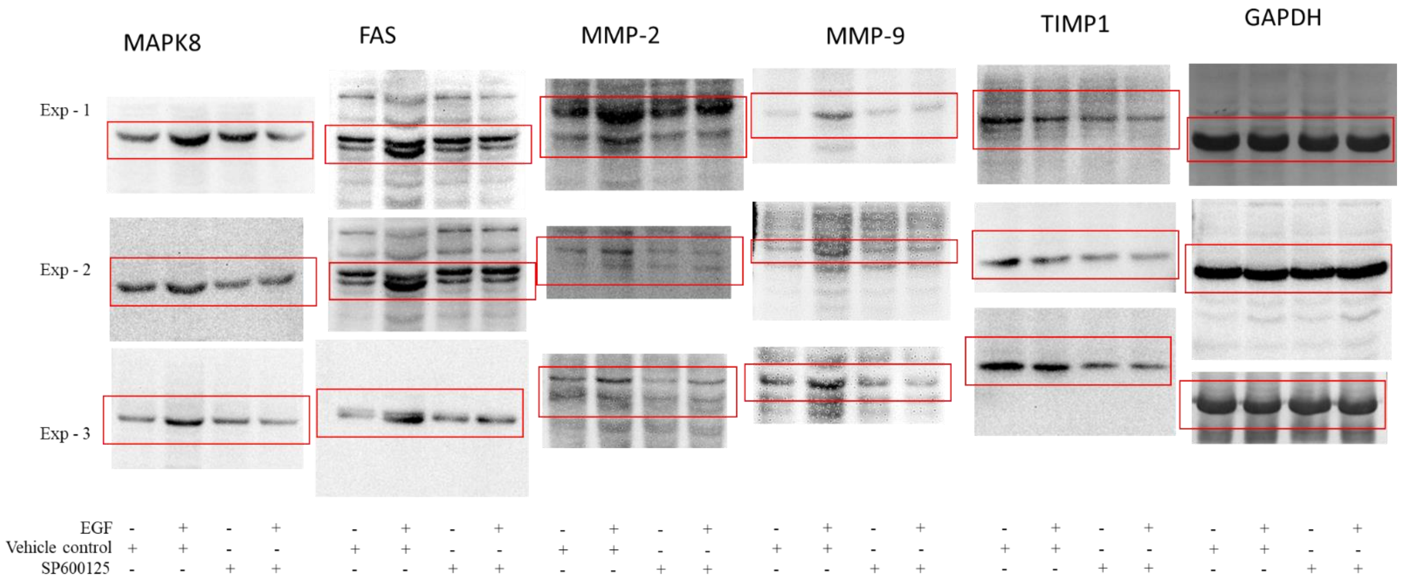

Fig. S10 - Effect of MAPK8 inhibitor (SP600125) on activation of MAPK8, FAS, MMP-2, MMP-9 and TIMP1 in EGF treated HTR-8/SVneo cells. Cell lysates were prepared from HTR-8/SVneo cells pretreated with SP600125 and control cells with or without EGF treatment, and Western blotting performed for determining MAPK8, FAS, MMP-2, MMP-9, TIMP1 and GAPDH protein expression as described in *Methods*. Blots from three independent experiments are shown.

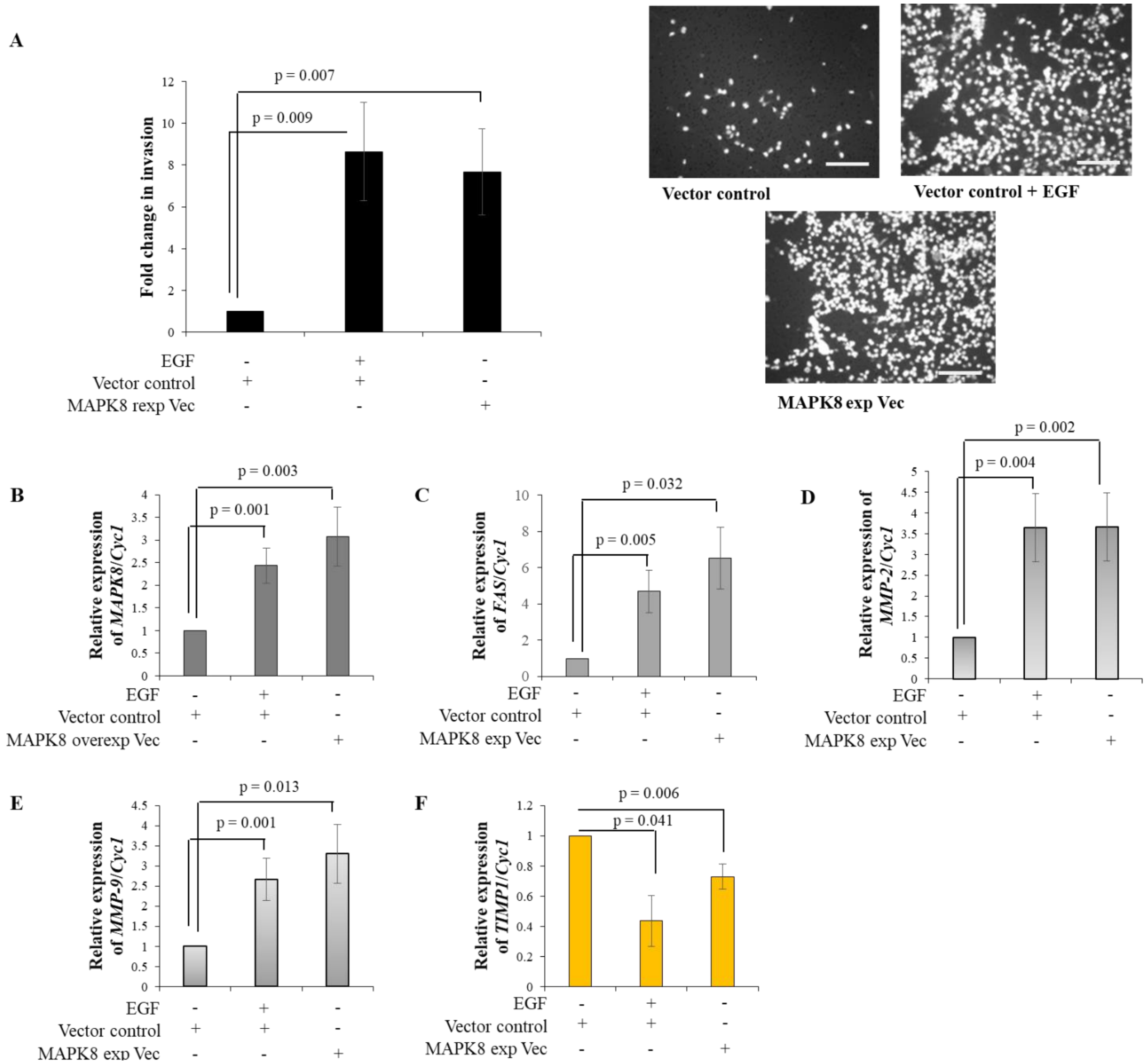

Fig. S11 - MAPK8 overexpression increases HTR-8/SVneo cell invasion by activation of MMP-2 and MMP-9. HTR-8/SVneo cells transfected with mammalian MAPK8 expression vector were used to perform Matrigel invasion assay as well as expression of *MAPK8*, *FAS*, *MMP-2*, *MMP-9* and *TIMP1* after 24 h treatment. Panel (A) the bar graph shows fold change in invasion of EGF treated and MAPK8 transfected HTR-8/SVneo cells as compared to vector control transfected HTR-8/SVneo cells. Values are expressed as mean ± SEM of three independent experiments performed in duplicates. Representative photographs of invading cells in various treatment groups on 0.8 µm pore size transwell membranes as observed under microscope after processing for invasion assay are appended alongside. Scale bar represents 5 µm. Relative expression profiles at transcript levels of *MAPK8* (B), *FAS* (C), *MMP-2* (D), *MMP-9* (E) and

TIMP1 (F) with respect to *Cyc1* as loading controls in MAPK8 mammalian expression vector transfected HTR-8/SVneo cells as well as those treated with EGF (10 ng/ml) as compared to vector control transfected cells are also shown. The transcript data is shown as mean ± SEM of three independent experiments performed in triplicates.

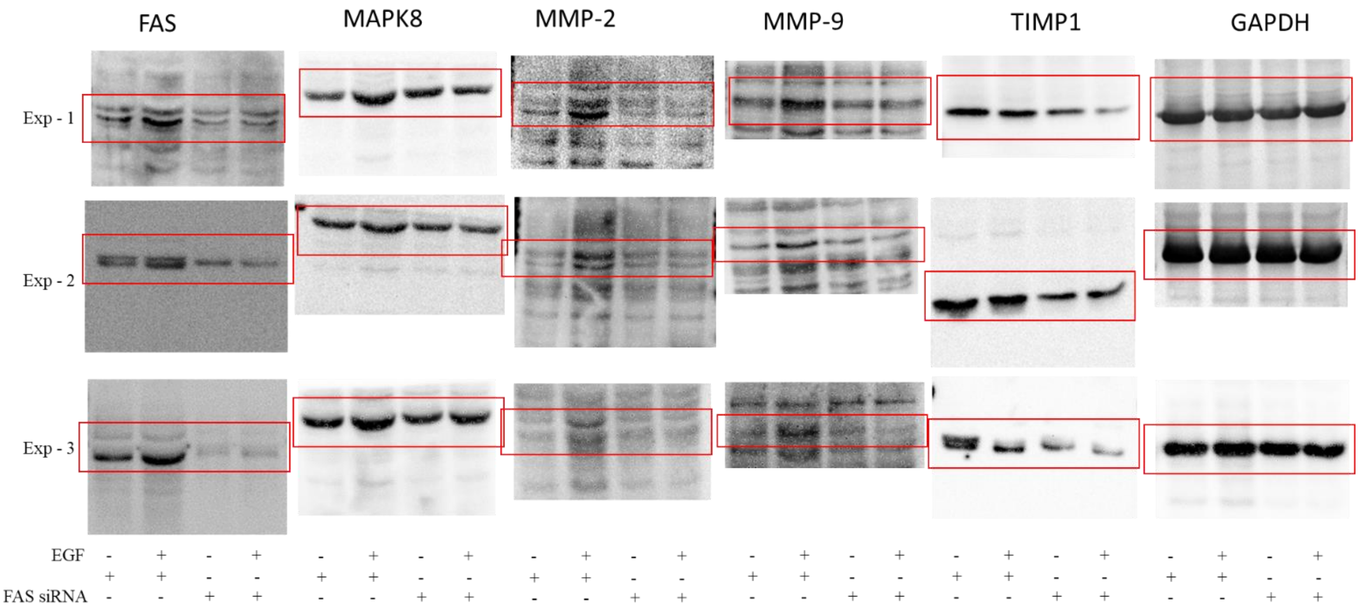

Fig. S12 - Effect of FAS silencing on activation of FAS, MAPK8, MMP-2, MMP-9 and TIMP1 in EGF treated HTR-8/SVneo cells. Cell lysates were prepared from HTR-8/SVneo cells transfected with FAS and control siRNAs respectively with or without EGF treatment, and Western blotting performed for determining FAS, MAPK8, MMP-2, MMP-9, TIMP1 and GAPDH protein expression as described in *Methods*. Blots from three independent experiments are shown.

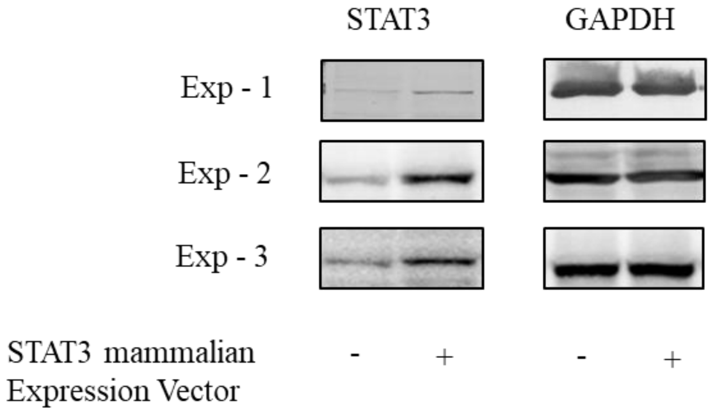

Fig. S13 – Increased expression of total STAT3 in HEK-293T cells transfected with STAT3 mammalian expression vector. Western blotting was performed from the cell lysates of untreated and STAT3 mammalian expression vector transfected HEK-293T cells after 24 h of transfection. Blots of total STAT3 and GAPDH (as an internal control) of three independent experiments are shown confirming the increased expression of STAT3 in STAT3 mammalian expression vector transfected HEK-293T cells.

Table S1 – Sequence of the synthetically synthesized sense and antisense oligos

Name	Sequence 5' to 3'	Nt
**MAPK8 WT sense**	TCGAGCTGTATCATTTATTGTAAATGCCAGCTGCCACTTGCCAACCATCATGTTCAGTG	59
**MAPK8 WT antisense**	TCGACACTGAACATGATGGTTGGCAAGTGGCAGCTGGCATTTACAATAAATGATACAGC	59
**MAPK8 Mut sense**	TCGAGCTGTATCATTTATTGTAAATGCCAGCTGCCACTTGAAAAAAATCATGTTCAGTG	59
**MAPK8 Mut antisense**	TCGACACTGAACATGATTTTTTTCAAGTGGCAGCTGGCATTTACAATAAATGATACAGC	59
**FAS WT sense**	TCGAGGGTGATGTGGTTTTTTTCCTCATGGCTTCACCTAGTGGCCCCAAGCATGACTTG	59
**FAS WT antisense**	TCGACAAGTCATGCTTGGGGCCACTAGGTGAAGCCATGAGGAAAAAAACCACATCACCC	59
**FAS Mut sense**	TCGAGGGTGATGTGGTTTTTTTCCTCATGGCTTCACCTAGTGGCAAAAAGCATGACTTG	59
**FAS Mut antisense**	TCGACAAGTCATGCTTTTTGCCACTAGGTGAAGCCATGAGGAAAAAAACCACATCACCC	59
**STAT3 BS1 sense**	CGCGTTTTAAACCAATTTATAATTGCTGTATTTCTCGTAAATGTTATGAATCCTCACTTTTAACAATTA	69
**STAT3 BS1 antisense**	GATCTAATTGTTAAAAGTGAGGATTCATAACATTTACGAGAAATACAGCAATTATAAATTGGTTTAAAA	69
**STAT3 BS2 sense**	CGCGTCACGCGGCCAGGTTAAGGAAACACATTGCCACGAAGTGTGCATTCACAAGAGTGTCGGGTGTAA	69
**STAT3 BS2 antisense**	GATCTTACACCCGACACTCTTGTGAATGCACACTTCGTGGCAATGTGTTTCCTTAACCTGGCCGCGTGA	69

WT - wild type binding site

Blue color – wild type nucleotides

Mut - mutated binding site

Red color – mutated nucleotides

BS1 – binding site 1 (green color)

BS2 – binding site 2 (purple color)

Table S2 – Relative expression of MMPs and TIMPs in EGF treated HTR-8/SVneo cells as compared to untreated control cells

Gene	Relative ΔCt of the transcript levels in EGF treated as compared to untreated HTR-8/SVneo cells at 24 h		p value
	Untreated	EGF	
MMP-1	1 ± 0.01	1.65 ± 0.21	0.0765
MMP-2	1 ± 0.01	2.64 ± 0.33	0.0059
MMP-3	1 ± 0.01	0.94 ± 0.09	0.1288
MMP-7	1 ± 0.01	1.02 ± 0.01	0.0800
MMP-9	1 ± 0.01	1.90 ± 0.29	0.0068
MMP-12	1 ± 0.01	1.10 ± 0.02	0.2001
MMP-15	1 ± 0.01	1.09 ± 0.09	0.0729
MMP-16	1 ± 0.01	1.18 ± 0.75	0.1112
MMP-21	1 ± 0.01	0.89 ± 0.08	0.0603
MMP-23b	1 ± 0.01	0.82 ± 0.15	0.0556
TIMP1	1 ± 0.01	0.51 ± 0.14	0.0383
TIMP2	1 ± 0.01	0.81 ± 0.08	0.0581
TIMP3	1 ± 0.01	0.89 ± 0.10	0.1907
TIMP4	1 ± 0.01	0.96 ± 0.07	0.1382

Table S3 - Putative STAT1 and STAT3 binding sites in the promoter region of miR-92a-1-5p precursor encoding region

Micro RNA	Transcription Factor	No of binding sites	Position from miRNA precursor encoding region	Binding Characteristics	Sequence of the binding site
**mir-92a-1-5p**	STAT1	3	1010-1028	Homo/Hetero-dimer	gggctcccaGGAAgccccg
			1035-1053	Homo/Hetero-dimer	caccctcctGGAAgcgcac
			3441-3459	Homo-dimer	cccattccaGAAAacttcc
	STAT3	4	492-510	Homo-dimer	gtatTTCTcgtaaatgtta
			777-795	Homo-dimer	acatTGCCacgaagtgtgc
			1008-1026	Homo/Hetero-dimer	gggcTTCCtgggagccctg
			1037-1055	Hetero-dimer	gcgcTTCCaggagggtgtg

Table S4 - Primers of genes and miRNAs for qRT-PCR

Target	Primer pair (5’ - 3’)	Annealing temperature
miR-92a-1-5p	FP: CGC AGA GGT TGG GAT CGG TTG<br>RP: CAG GTC CAG TTT TTT TTT TTT TTT AGC ATTG	60°C
miR-19a-5p	FP: GCA GGT TTT GCA TAG TTG CAC<br>RP: GGT CCA GTT TTT TTT TTT TTT TTG TAG	60°C
miR-191	FP: CGC AGC AAC GGA ATC CCA AAA G<br>RP: CAG GTC CAG TTT TTT TTT TTT TTT CAG CTG	60°C
MAPK8	FP: ATGAAGCTCTCCAACACCCG<br>RP: TGTGCTAAAGGAGAGGGCTG	60°C
FAS	FP: ACTGTGACCCTTGCACCAAA<br>RP: AGACAAAGCCACCCCAAGTT	60°C
TCF7	FP: CAAGCAGAGTCCAAGGCAGA<br>RP: TGTGGTGGATTCTTGGTGCT	60°C
CD151	FP: CCTAGAGTCCTGGGGAGCTT<br>RP: CACCAGGATGTAGGCTGTGG	60°C
CYC1	FP: AGAGTTTGACGATGGCACCCC<br>RP: GCCTCCCAACCCTTTTACCTT	60°C
MMP-1	FP: CCCATCGGCCCACAAACCCC<br>RP: AGCAGCTTCAAGCCCATTTGGCA	60°C
MMP-2	FP: ACCGCAAGTGGGGCTTCTGC<br>RP: CGTGGCCAAACTCGTGGGCT	59°C
MMP-3	FP: TTGGCCCATGCCTATGCCCC<br>RP: ACAGGCGGAACCGAGTCAGG	60°C
MMP-7	FP: GTCTCTGGACGGCAGCTATG<br>RP: GATAGTCCTGAGCCTGTTCCC	60°C
MMP-9	FP: CCGGCATTCAGGGAGACGCC<br>RP: TGGAACCACGACGCCCTTGC	60°C
MMP-12	FP: ACATTTCGCCTCTCTGCTGGATGAC<br>RP: CAGAAACCTTCAGCCAGAAGAACC	60°C
MMP-15	FP: AGAACTGGCTGCGGCTTTAT<br>RP: CGCTTCATCCACTCCTTGGT	60°C
MMP-16	FP: TGTACCTGACCAGACAAGAG<br>RP: AGTGTCCATGGCTCATCTGA	58°C
MMP-21	FP: TCCACGCTGCTCAGCGGT<br>RP: TCCACGCTGCTCAGCGGT	60°C
MMP-23b	FP: GCTGGTCGCCCTGTGCCTC<br>RP: GGAGTCAGGCGTGTAGCGGCG	60°C
TIMP1	FP: TGACATCCGGTTCGTCTACA<br>RP: GTTTGCAGGGGATGGATAAA	57°C
TIMP2	FP: GATGCACATCACCCTCTGTG<br>RP: GTGCCCGTTGATGTTCTTCT	58°C
TIMP3	FP: CTGACAGGTCGCGTCTATGA<br>RP: AGTCACAAAGCAAGGCAGGT	58°C
TIMP4	FP: TGGGTGAGGCATGCAGCTGC<br>RP: GGGTCTGCACTGGCCGGAAC	57°C
